# Entropy Enhanced Perovskite Oxide Ceramic for Efficient Electrochemical Reduction of Oxygen to Hydrogen Peroxide

**DOI:** 10.1002/anie.202200086

**Published:** 2022-03-23

**Authors:** Ziliang Chen, Jie Wu, Zhengran Chen, Hongyuan Yang, Kai Zou, Xiangyong Zhao, Ruihong Liang, Xianlin Dong, Prashanth W. Menezes, Zhenhui Kang

**Affiliations:** ^1^ Institute of Functional Nano and Soft Materials (FUNSOM) Jiangsu Key Laboratory for Carbon-based Functional Materials and Devices Joint International Research Laboratory of Carbon-Based Functional Materials and Devices Soochow University Suzhou 215123 China; ^2^ Department of Chemistry: Metalorganics and Inorganic Materials Technische Universität Berlin Straße des 17 Juni 135, Sekr. C2 10623 Berlin Germany; ^3^ Key Laboratory of Inorganic Functional Materials and Devices Shanghai Institute of Ceramics Chinese Academy of Sciences 588 Heshuo Road, Jiading District Shanghai 201800 China; ^4^ Material Chemistry Group for Thin Film Catalysis—CatLab Helmholtz-Zentrum Berlin für Materialien und Energie Albert-Einstein-Str. 15 12489 Berlin Germany; ^5^ Key Laboratory of Optoelectronic Material and Device Department of Physics Shanghai Normal University Shanghai 200234 China

**Keywords:** Electrocatalysis, High Entropy, Hydrogen Peroxide, Oxygen Reduction Reaction, Perovskite Oxide Ceramic

## Abstract

The electrochemical oxygen reduction reaction (ORR) offers a most promising and efficient route to produce hydrogen peroxide (H_2_O_2_), yet the lack of cost‐effective and high‐performance electrocatalysts have restricted its practical application. Herein, an entropy‐enhancement strategy has been employed to enable the low‐cost perovskite oxide to effectively catalyze the electrosynthesis of H_2_O_2_. The optimized Pb(NiWMnNbZrTi)_1/6_O_3_ ceramic is available on a kilogram‐scale and displays commendable ORR activity in alkaline media with high selectivity over 91 % across the wide potential range for H_2_O_2_ including an outstanding degradation property for organic dyes through the Fenton process. The exceptional performance of this perovskite oxide is attributed to the entropy stabilization‐induced polymorphic transformation assuring the robust structural stability, decreased charge mobility as well as synergistic catalytic effects which we confirm using advanced *in situ* Raman, transient photovoltage, Rietveld refinement as well as finite elemental analysis.

## Graphical abstract

A high‐entropy perovskite oxide ceramic Pb(NiWMnNbZrTi)_1/6_O_3_ electrocatalyst has been synthesized on a kilogram‐scale *via* the thermal‐induced solid solution method that delivers efficient H_2_O_2_ production by an oxygen reduction reaction and spontaneously degrades organic dyes through the Fenton reaction.

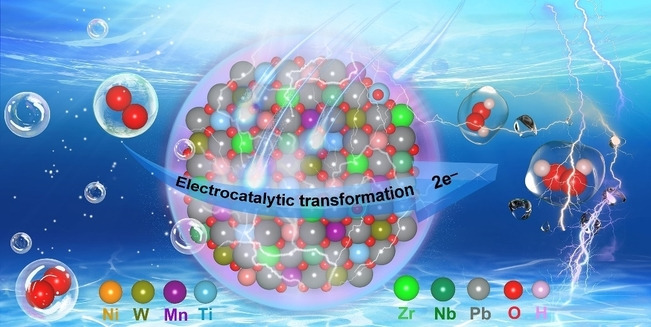

## Introduction

Hydrogen peroxide (H_2_O_2_) is one of the most significant and fundamental industrial chemicals for several frontier fields including environment, agriculture, and energy.^[1]^ Currently, the World Health Organization (WHO) has listed H_2_O_2_ as the crucial disinfectant against SARS‐CoV‐2 that causes the COVID‐19 pandemic.^[2]^ The generally adopted industrial method for large‐scale H_2_O_2_ production is the anthraquinone cycling process.^[3]^ However, this process demands straightforward participation of hydrogen and oxygen gases coupled with catalysis using expensive palladium, followed by a tedious separation procedure to limit the substantial amount of organic wastes.^[4]^ In this regard, it is crucial to develop an alternative method to synthesize H_2_O_2_ by decreasing energy consumption, production cost, and waste generation.

In recent years, the electrochemical synthesis of H_2_O_2_ from the oxygen reduction reaction (ORR) through the 2 e^−^ transfer process has aroused great interest as it can theoretically overcome all the drawbacks which are parasitic in the traditional anthraquinone method.^[5]^ Nevertheless, ORR is a competitive reaction and favors a 4 e^−^ pathway to form water molecules.^[6]^ Therefore, developing an electrocatalyst featuring high activity and selectivity towards a 2 e^−^ pathway is the prerequisite for the sustainable electrosynthesis of H_2_O_2_ *via* ORR.^[7]^ In this context, several carbonaceous materials, transition metal‐based compounds as well as their hybridization have been investigated as advanced electrocatalysts for the electrosynthesis of H_2_O_2_.^[8]^ Nevertheless, most of these electrocatalysts can still only be produced at the (milli)gram level, which is likely to restrict the large‐scale application. Besides, the preparation procedures for such electrocatalysts are usually demanding as they require a rigorous coordination chemistry environment, control of the morphology as well as inhibition of (nano)particles agglomeration.

Moreover, most of the reported electrocatalysts in the catalytic process undergo severe oxidation at high operation voltage, and the overpotential window to attain a sustained high peroxide selectivity is narrow (≈0.4 V), thus inevitably leading to poor durability and efficiency.[^5a^, ^9^] Although continuous progress has been achieved over the years to develop catalysts with high efficiency for the electrosynthesis of H_2_O_2_, there are still several formidable challenges to enabling an electrocatalyst that can be readily prepared in large quantities to economically convert O_2_ to H_2_O_2_ over a broad voltage range whilst concurrently maintaining robust stability and yield.

Perovskite oxides are a category of complex oxides with the chemical formula *AB*O_3_, in which *A* atoms have larger cation radii than those of *B* atoms that are surrounded by the network of corner‐sharing *B*O_6_ octahedra.^[10]^ The unique crystal structure combined with the advantages of tunable composition and low cost render them highly promising for efficient electrocatalysts.^[11]^ Previous studies have demonstrated that perovskite oxides are capable of effectively catalyzing the 4 e^−^ ORR, showing their potential for fuel cells.^[12]^ Cho et al. reported that the catalyst based on La_0.3_(Ba_0.5_Sr_0.5_)_0.7_Co_0.8_Fe_0.2_O_3−*δ*_ delivered a four‐electron transfer pathway, however, in the absence of La, the electron transfer number of Ba_0.5_Sr_0.5_Co_0.8_Fe_0.2_O_3−*δ*_ was decreased to 3.2, which meant that the 2 e^−^ ORR pathway began to compete during ORR reaction.^[13]^ From this report, it can be concluded that the 2 e^−^ pathway for ORR is feasible with perovskite oxide electrocatalysts albeit the selectivity is unsatisfactory. This shows that it is indeed possible to drive a 2 e^−^ pathway‐dominated ORR by tuning the composition and structure of perovskite oxides, yet the related investigation is still lacking. On the other hand, high‐entropy materials (alloys, oxides, and sulfides) have recently received extensive interest as novel electrocatalysts for a variety of catalytic applications including hydrogen evolution reaction, oxygen evolution reaction, and 4 e^−^ pathway‐dominated ORR.^[14]^ These high‐entropy materials can be synthesized by several appealing entropy‐driven strategies.^[15]^ Owing to the intrinsic traits of tunable composition, cocktail effect, as well as high corrosion and oxidation resistance, all of these high‐entropy electrocatalysts showed impressive catalytic activity. Therefore, based on the premises, combining perovskite oxide with the concept of high‐entropy to form high‐entropy perovskite oxides might be an effective approach to realize high‐efficiency 2 e^−^ ORR.

Bearing the above points in mind, herein, a high‐entropy perovskite oxide ceramic Pb(NiWMnNbZrTi)_1/6_O_3_ derived from the Pb(ZrTi)_1/2_O_3_ prototype was deliberately synthesized *via* the thermal‐induced solid solution method for the efficient H_2_O_2_ production by ORR. Here, the metal species in the *B* site are expected to be able to drive 2 e^−^ ORR, while the inert Pb in *A* site is anticipated to serve as a barrier to isolate the surrounding *B* atom and thereby promotion of 2 e^−^ ORR.^[16]^ Strikingly, by combining *in situ* Raman, *ex situ* transient photovoltage measurement (TPV) with Rietveld refinement and finite element analysis (FEA), we disclose that upon the construction of high‐entropy, the crystal structure transforms from the initial tetragonal‐type Pb(ZrTi)_1/2_O_3_ to the cubic‐type Pb(NiWMnNbZrTi)_1/6_O_3_, showing a decreased internal lattice strain. Meanwhile, the presence of high‐entropy decreases the charge transfer ability and optimizes the surface charge density, contributing to the selectivity towards 2 e^−^ ORR. Moreover, all *B*‐site metal species could serve as the catalytic active center and synergistically perform catalysis. Benefiting from these merits, the as‐synthesized Pb(NiWMnNbZrTi)_1/6_O_3_ exhibited a selectivity as high as 96 % at the potential of 0.4 V *vs*. RHE. In addition, it also showed negligible performance decay even after 12 h of duration at 0.4 V *vs*. RHE, together with an outstanding dye degradation property for methylene blue (MB), methyl orange (MO), and rhodamine B (RB). The performances attained here are not only significantly better than those of Pb(ZrTi)_1/2_O_3_ but also one of the best among previously reported electrocatalysts. Most importantly, the synthetic strategy adopted here for the preparation of catalysts can easily be scaled‐up (to the kilogram‐level) and has huge potential for future practical applications.

## Results and Discussion

### Synthesis, Phase, and Microstructural Features

**Figure 1:**
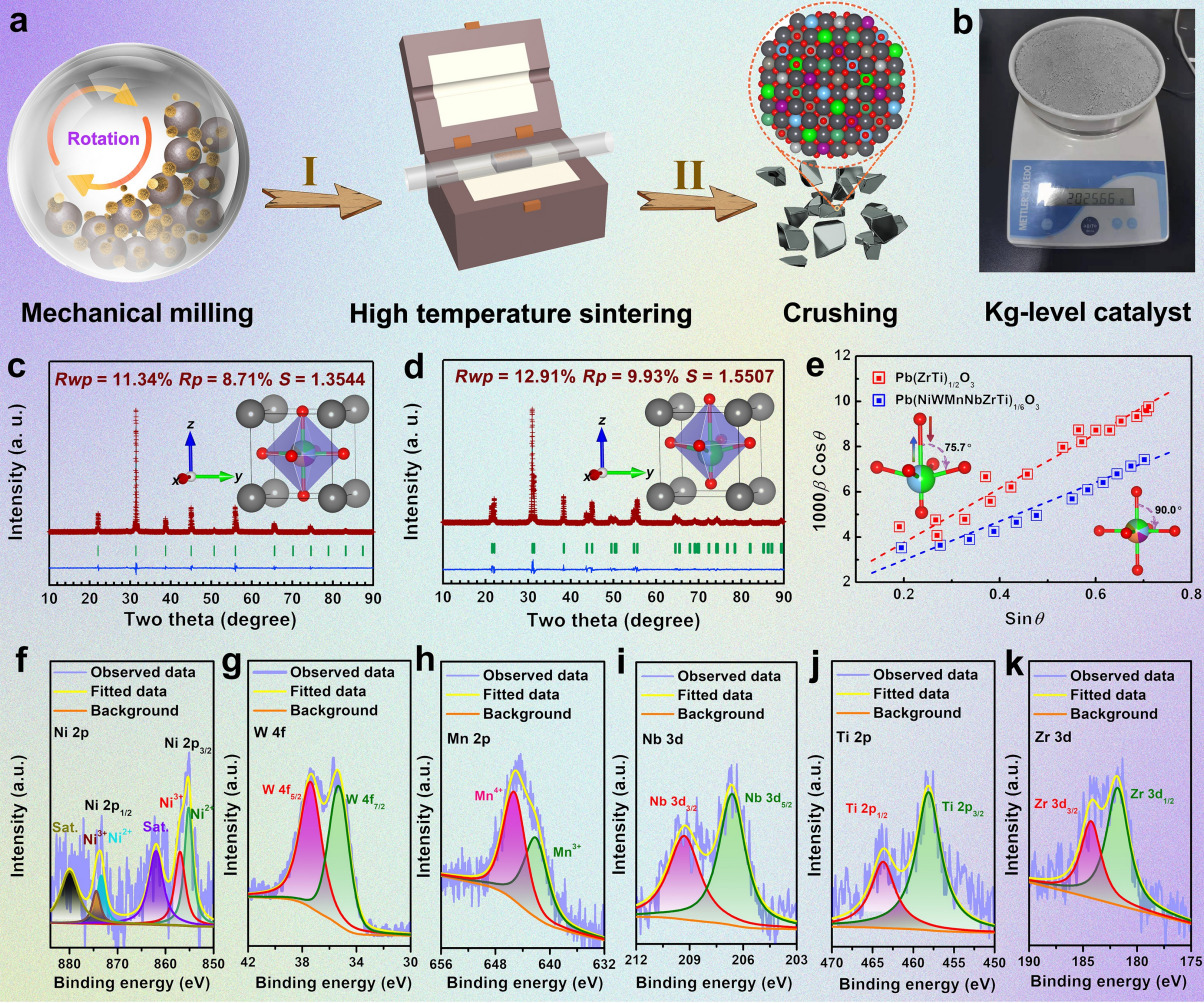
a) Schematic synthesis of high‐entropy ceramic Pb(NiWMnNbZrTi)_1/6_O_3_ compound. b) Digital image of the kilogram‐level production of Pb(NiWMnNbZrTi)_1/6_O_3_. Rietveld refinement of XRD patterns for c) Pb(NiWMnNbZrTi)_1/6_O_3_ and d) Pb(ZrTi)_1/2_O_3_. e) Comparison of lattice strain of Pb(NiWMnNbZrTi)_1/6_O_3_ and d) Pb(ZrTi)_1/2_O_3_. Green line, red “+” symbol, blue line, and green vertical bars showed in Figures (c) and (d) represent the observed data, fitted data, differentiation, and the positions of the reflection peaks of the phase in the XRD patterns. High‐resolution XPS spectra of f) Ni 2p, g) W 4f, h) Mn 2p, i) Nb 3d, j) Ti 2p, and k) Zr 3d in Pb(NiWMnNbZrTi)_1/6_O_3_.

Figure 1a schematically illustrates the synthesis procedure for the high‐entropy perovskite oxide. First, target metal oxides and carbonate powders were mixed homogeneously by high‐energy ball‐milling. Then, the mechanical‐treated powders were pressed as pellets for high‐temperature sintering under the air to obtain the Pb(NiWMnNbZrTi)_1/6_O_3_ ingots. Finally, the as‐prepared ingots were crushed into powders for further use. Remarkably, as visually presented in Figure 1b, the catalyst could be prepared up to a kilogram‐scale only by a one‐batch operation. The inductively coupled plasma mass spectrometry (ICP‐MS) indicated that the atomic ratio of Pb : Ni : W : Mn : Nb : Zr : Ti was 1 : 0.164 : 0.165 : 0.160 : 0.163 : 0.174 : 0.174 (Table S1), which was quite close to the designed composition, demonstrating the successful preparation of the Pb(NiWMnNbZrTi)_1/6_O_3_ by thermal‐solid diffusion. Rietveld refinement of the X‐ray powder diffraction pattern (XRD) showed a pure perovskite oxide phase crystallizing in cubic structure (*Pm* $\bar{3}$ *m*) with refined lattice parameters *a*=*b*=*c*=4.0245(4) Å (Figure 1c, Table S2, and S3). For comparison, the pure Pb(ZrTi)_1/2_O_3_ was also synthesized under the same condition (Figure 1d, Table S1). Interestingly, the Pb(ZrTi)_1/2_O_3_ phase was crystallized in the tetragonal structure with lower symmetry (*P*4*mm*) (Table S2 and S4). Moreover, based on the Williamson‐Hall method, the lattice strain for Pb(NiWMnNbZrTi)_1/6_O_3_ (+1.05(5)%) was found to be much smaller as compared to that (+1.60(4)%) of Pb(ZrTi)_1/2_O_3_ (Figure 1d). Such results clearly indicated that an entropy enhancement could induce the release of lattice strain and led to the polymorphic phase transformation of Pb(ZrTi)_1/2_O_3_, which was expected to improve the structural stability. Figure 1f–k reveals the high‐resolution X‐ray photoelectron spectroscopy (XPS) spectra of Ni 2p, W 2p, Mn 2p, Nb 2p, Zr 2p, and Ti 2p, respectively, where their respective oxidation states have been shown. Specifically, it could be seen from Figure 1f that Ni 2p_1/2_ and Ni 2p_3/2_ were distributed at 873.7 and 855.3 eV, respectively, both of which could be deconvoluted into Ni^3+^ and Ni^2+^, suggesting the presence of Ni−O bond.^[17]^ The peaks at 37.4 and 35.4 eV in Figure 1g could be assigned to W 4f_5/2_ and W 4f_7/2_, respectively, which were widely identified as W^6+^ in WO_3_.^[18]^ The spectrum displayed in Figure 1h could be divided into two peaks at 645.4 and 642.2 eV, which were attributed to the Mn^4+^ and Mn^3+^, respectively.^[19]^ As presented in Figure 1i, two characteristic peaks at 209.3 and 206.6 eV could be ascribed to Nb 3d_3/2_ and Nb 3d_5/2_ in the niobium oxide.^[20]^ Moreover, the Ti−O,^[21]^ Zr−O,^[22]^ and Pb−O^[23]^ bonds could also be observed in the high‐resolution Ti 2p (Figure 1j), Zr 3d (Figure 1k), and Pb 4 f (Figure S1a) XPS spectra, respectively. The high‐resolution O 1s XPS spectra further confirmed the presence of metal‐O bonds (Figure S1b).^[24]^ For comparison, the XPS spectra for all elements in Pb(ZrTi)_1/2_O_3_ are shown in Figure S2. The comparative results demonstrated that the entropy enhancement could effectively modify the electronic structure of the compound (For details refer to Figure S2).

**Figure 2:**
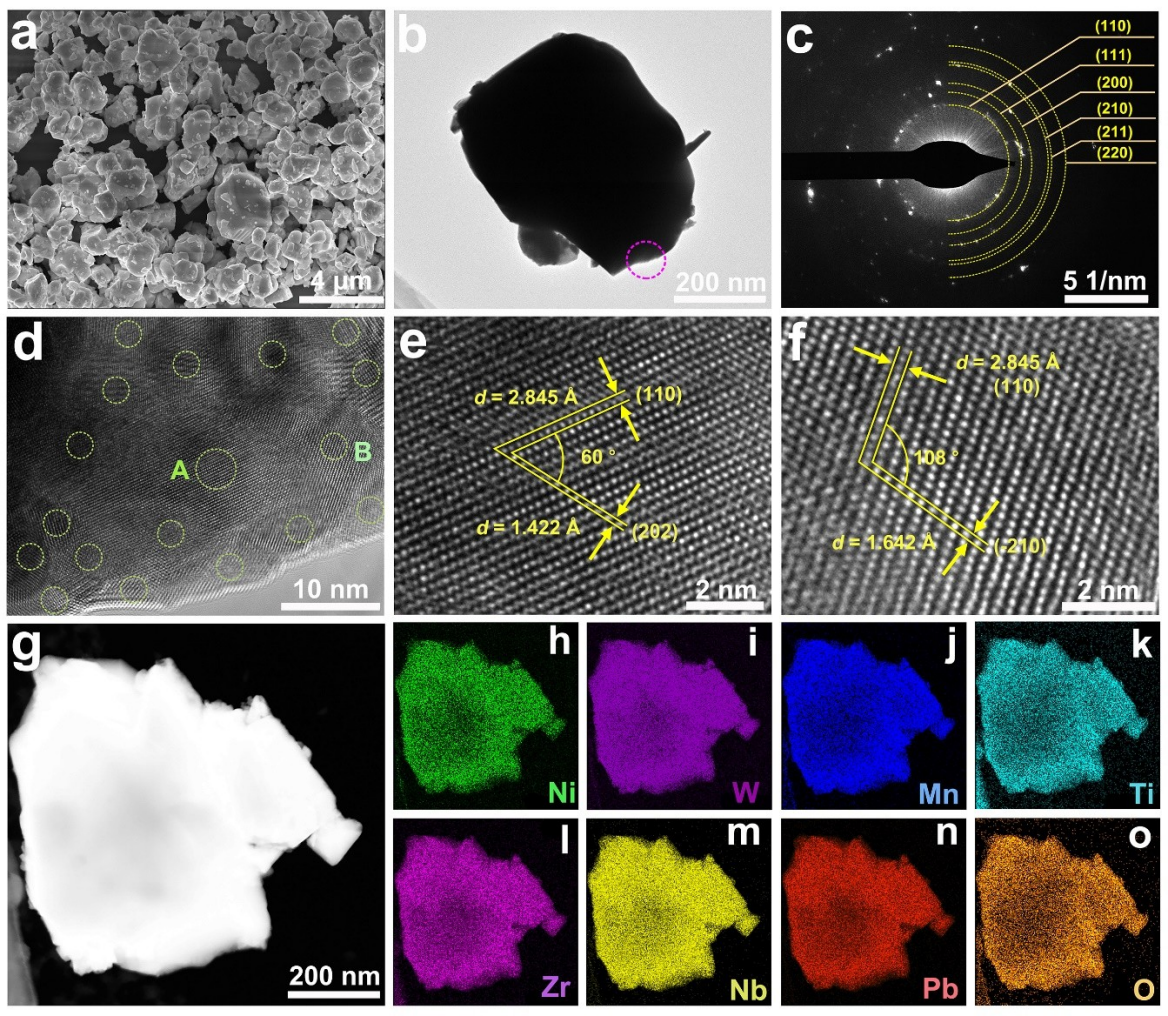
a) FESEM image, b) TEM image; c) corresponding SAED pattern, d) high‐magnified TEM image, e, f) high‐resolution TEM images, g) HAADF image of representative Pb(NiWMnNbZrTi)_1/6_O_3_ particle and the corresponding elemental mapping of h) Ni, i) W, j) Mn, k) Ti, l) Zr, m) Nb, n) Pb, and o) O.

To further acquire the microstructure information, the field emission scanning electron microscopic (FESEM) image for Pb(NiWMnNbZrTi)_1/6_O_3_ was conducted (Figure 2a) that exhibited uniform dispersion of pseudosphere particles with sizes ranging from nanoscale to microscale. Subsequently, the transmission electron microscopic (TEM) image of the representative Pb(NiWMnNbZrTi)_1/6_O_3_ particle was recorded (Figure 2b). The selected area electron diffraction (SAED) pattern of the chosen region of the particle (Figure 2b) showed diffraction rings/dots that can be precisely indexed to (110), (111), (200), (210), (211) and (220) facets of Pb(NiWMnNbZrTi)_1/6_O_3_ phase (Figure 2c), respectively. Moreover, the result obtained from SAED is in good agreement with the XRD pattern. The magnified TEM image displayed in Figure 2d shows a number of nanocrystallites (as marked by the dotted circle). High‐resolution TEM (HRTEM) images for the region A and B of Figure 2d are presented in Figure 2e and 2 f, respectively, in which (110) and (202) facets with an intersection angle of about 60° and (110) and (−210) facets with an intersection angle of about 108° belonging to Pb(NiWMnNbZrTi)_1/6_O_3_ phase were confirmed. In addition, the high angle annular dark‐field scanning TEM (HAADF‐STEM) pattern exhibited the bright region indicating the homogeneous distribution of metal species. The corresponding EDS mapping further substantiated the high compositional uniformity of the Pb(NiWMnNbZrTi)_1/6_O_3_ phase within the entire particle (Figure 2g–o). Besides, the phase purity, structure, and composition of Pb(ZrTi)_1/2_O_3_ were also verified by the HRTEM and EDS mapping results (Figure S3).

### Effect of Entropy on ORR Activity

**Figure 3:**
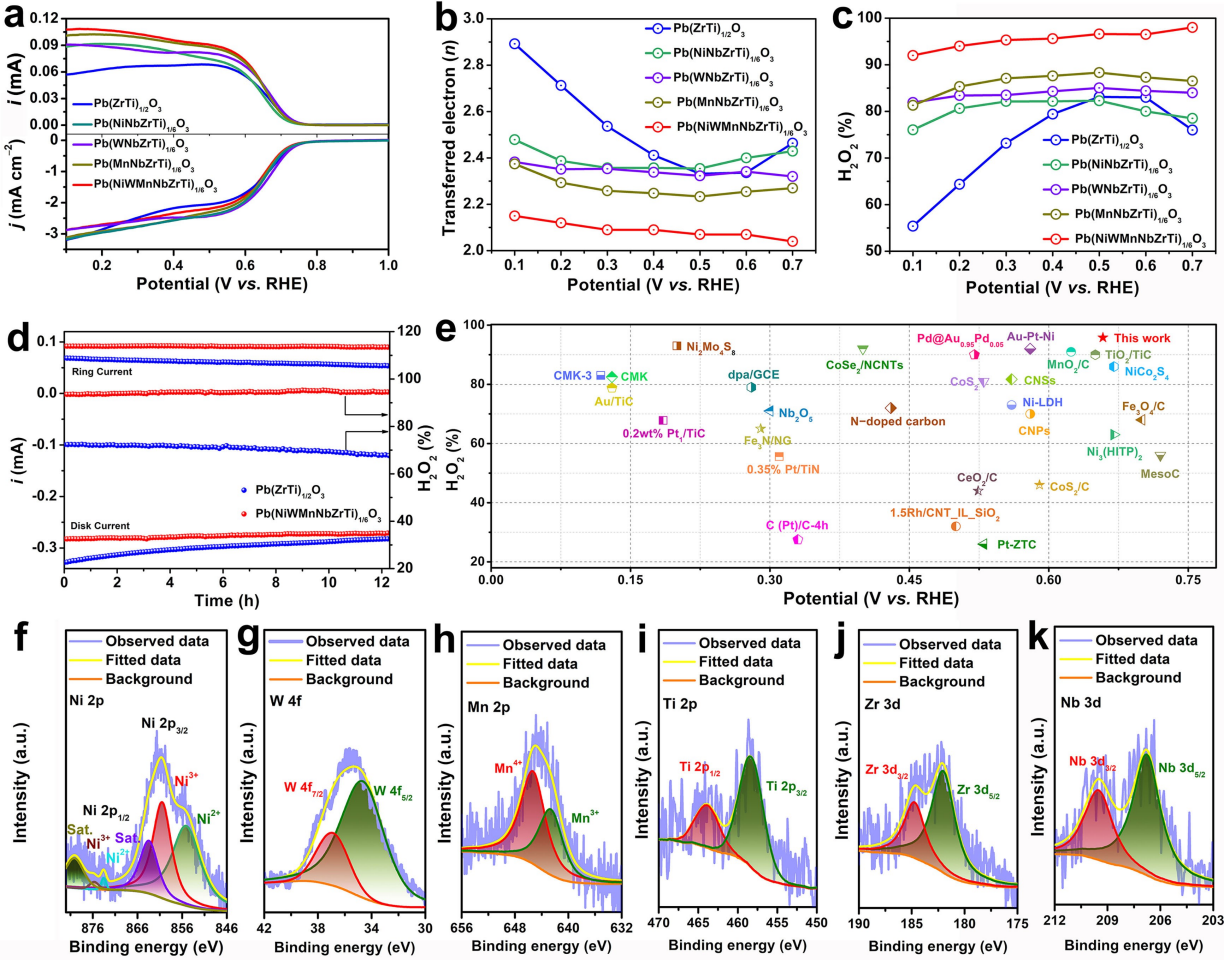
a) LSV curves of Pb(NiWMnNbZrTi)_1/6_O_3_, Pb(ZrTi)_1/2_O_3_, Pb(MnNbZrTi)_1/4_O_3_, Pb(WNbZrTi)_1/4_O_3_, and Pb(NiNbZrTi)_1/4_O_3_ recorded at 1600 rpm and 10 mV s^−1^ (bottom part), together with the corresponding H_2_O_2_ current on the ring electrode (upper part). b) Calculated electron transfer number and c) selectivity of H_2_O_2_ within the potential sweep. d) Comparison of stability of Pb(NiWMnNbZrTi)_1/6_O_3_ and Pb(ZrTi)_1/2_O_3_ at a fixed disk potential of 0.1 V. e) Comparison of ORR activity of Pb(NiWMnNbZrTi)_1/6_O_3_ with previously reported electrocatalysts; High‐resolution XPS spectra of f) Ni 2p, g) W 4f, h) Mn 2p, i) Ti 2p, j) Zr 3d, and k) Nb 3d in Pb(NiWMnNbZrTi)_1/6_O_3_ after the chronoamperometry test.

Motivated by the unique structure and composition, the electrochemical ORR activity of Pb(NiWMnNbZrTi)_1/6_O_3_ to produce H_2_O_2_ was systematically examined by employing the rotating ring disk electrode (RRDE) technique in 0.1 M KOH, together with PbZrO_3_, PbTiO_3_, Pb(ZrTi)_1/2_O_3_, Pb(MnNbZrTi)_1/4_O_3_, Pb(WNbZrTi)_1/4_O_3_, and Pb(NiNbZrTi)_1/4_O_3_ for comparison (Figure S4–S11). The collection efficiency of the RRDE was determined as 0.37 (Figure S12). Figure 3a provided the linear sweep voltammetry (LSV) curves of the electrocatalysts at the scan rate of 10 mV s^−1^, in which both the disk and ring current density are presented. Notably, the onset potentials at the disk and ring electrodes were both around ≈0.76 V *vs*. RHE for Pb(NiWMnNbZrTi)_1/6_O_3_, and the potential to deliver a high current density of 1.0 mA cm^−2^ was 0.66 V *vs*. RHE, showing the outstanding ORR catalytic activity. Moreover, according to the disk and ring current density, the average electron transfer number (*n*) towards ORR for all the electrocatalysts was calculated and compared in Figure 3b and Figure S13. As expected, the *n* for Pb(NiWMnNbZrTi)_1/6_O_3_ was calculated to be ≈2.08 in the potential range of 0.3–0.7 V *vs*. RHE, which was the lowest value among the tested electrocatalysts, substantiating a 2 e^−^‐dominated ORR pathway. Notably, the selectivity of H_2_O_2_ production for Pb(NiWMnNbZrTi)_1/6_O_3_ was estimated above 91 % within the potential range from 0.1 to 0.7 V *vs*. RHE (Figure 3c). To strengthen our claims, we also synthesized other high‐entropy perovskites with quinary and novenary *B*‐site metals based on the Pb(ZrTi)_1/2_O_3_ prototype, such as Pb(NiWMnNbTi)_1/5_O_3_ (Figure S4, Figure S14), Pb(NiWMnNbZr)_1/5_O_3_ (Figure S4, Figure S15) and Pb(NiWMnNbTiZrFeVMo)_1/9_O_3_ (Figure S4, Figure S16). By comparing to the 2 e^−^ ORR activity of Pb(NiWMnNbZrTi)_1/6_O_3_, it could be further confirmed that the 2 e^−^ ORR activity was gradually increased with the enhancement of entropy in our studied system within a certain entropy range (Figure S17). Moreover, the partial incorporation of La into A‐site metal of Pb(NiWMnNbZrTi)_1/6_O_3_ was also carried out and the achieved Pb_0.9_La_0.1_(NiWMnNbZrTi)_1/6_O_3_ compound still exhibited the comparable 2 e^−^ ORR activity to Pb(NiWMnNbZrTi)_1/6_O_3_ (Figure S18 and S19).

On the other hand, since the high durability is the prerequisite for the practical production of H_2_O_2_ by ORR, the long‐time stability measurement of Pb(NiWMnNbZrTi)_1/6_O_3_ was conducted *via* chronoamperometry at a potential of 0.1 V (Figure 3d). Remarkably, the activity remained unchanged for 12 h, demonstrating the robust electrochemical production of H_2_O_2_. However, compared to Pb(NiWMnNbZrTi)_1/6_O_3_, only 86 % of the retention rate for Pb(ZrTi)_1/2_O_3_ could be obtained after 12 h, implying the advantage of high entropy in improving the structural stability. Such exceptional ORR performance of Pb(NiWMnNbZrTi)_1/6_O_3_, in terms of selectivity and durability, surpasses most of the recently reported electrocatalysts thus ranking it among one of the best non‐precious‐metal electrocatalysts (Figure 3e, Table S5). In order to reveal the advantages of Pb(NiWMnNbZrTi)_1/6_O_3_ in the production of H_2_O_2_, we evaluated the decomposition degree of H_2_O_2_ on different catalysts (Figure S20). A certain amount of electrocatalysts were added into 0.1 M KOH containing 10 mM H_2_O_2_, and the Pb(NiWMnNbZrTi)_1/6_O_3_ possessed the lowest H_2_O_2_ decomposition among all the measured electrocatalysts. To further verify the highly selective H_2_O_2_ production, the determination of H_2_O_2_ was performed by the cerium sulfate (Ce^4+^) method. Upon gradually undergoing the process of Ce^4+^ titration, the color of the solution turned orange‐yellow. The theoretical concentration of H_2_O_2_ produced at 34 mA cm^−2^ for 1 h was calculated to be 864 mg L^−1^ whereas, in the real measurement, the H_2_O_2_ concentration achieved by the Ce^4+^ method was about 807 mg L^−1^, which was quite close to the theoretical value. The above results signified the high selectivity of Pb(NiWMnNbZrTi)_1/6_O_3_ towards H_2_O_2_ by ORR.

### Insights into the Entropy‐Improved ORR Activity

To acquire deeper insights into the impressive activity, selectivity, and durability, XPS was performed, and the composition of the Pb(NiWMnNbZrTi)_1/6_O_3_ after the above chronoamperometry test was examined. As seen in Figure 3f–k and Figure S21, the high‐resolution XPS spectra suggested that all chemical elements were preserved after the durability test. Moreover, after the chronoamperometry test, the binding energy for *B*‐site metal species of Ni 2p, Mn 2p, Ti 2p, Zr 3d, and Nb 3d was shifted to the higher energy by around 0.3 eV, while W 4 f shifted to lower energy by 0.3 eV. This difference could arise from the surface change during catalysis, suggesting that *B*‐sites were the main active sites for ORR.^[24]^ Note that the binding energy of high‐resolution XPS spectra for *A*‐site Pb was basically unchanged before and chronoamperometry test, implying its weak contribution to catalytic activity. These results were similar to those of Pb(ZrTi)_1/2_O_3_ after the chronoamperometry test (Figure S22). To further evaluate whether and how Pb(NiWMnNbZrTi)_1/6_O_3_ phase changed during the ORR process, the HRTEM, SAED, HAADF as well as the corresponding EDS mapping was recorded. As displayed in Figure S23, no new phase was identified after the chronoamperometry test and the attained results matched quite well with the initial Pb(NiWMnNbZrTi)_1/6_O_3_. However, some defects such as lattice distortion were more apparent on the surface of the particles, which could be ascribed to the catalysis‐induced surface reconstruction (Figure S23). Similar to the Pb(NiWMnNbZrTi)_1/6_O_3_, the HRTEM, SAED as well as HAADF of Pb(ZrTi)_1/2_O_3_ after chronoamperometry test also indicated the implicit surface reconstruction (Figure S24). To further compare the compositional stability, the element content in the 0.1 M KOH electrolyte after the chronoamperometry test was measured with ICP. The results obtained in Table S6 clearly revealed a higher element dissolution for Pb(ZrTi)_1/2_O_3_ than that of Pb(NiWMnNbZrTi)_1/6_O_3_.

**Figure 4:**
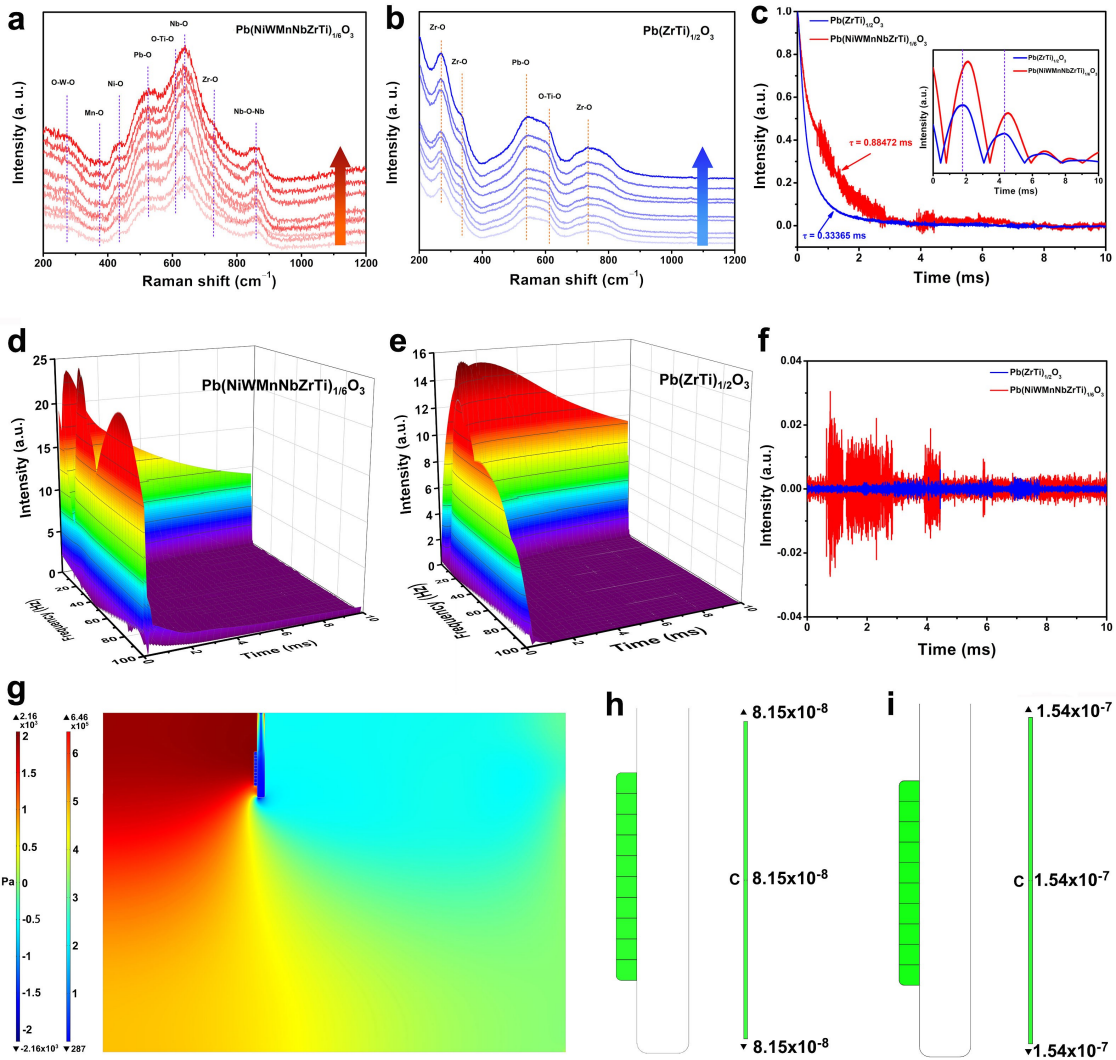
*In situ* Raman spectra of a) Pb(NiWMnNbZrTi)_1/6_O_3_ and b) Pb(ZrTi)_1/2_O_3_ in O_2_‐saturated 0.1 M KOH from −0.2 to −0.6 V *vs*. SCE with an interval voltage of 0.05 V. c) Comparison of TPV curves between Pb(NiWMnNbZrTi)_1/6_O_3_ and Pb(ZrTi)_1/2_O_3_ The inset in (c) shows the comparison of CWT curves between Pb(NiWMnNbZrTi)_1/6_O_3_ and Pb(ZrTi)_1/2_O_3_. 3D CWT patterns of d) Pb(NiWMnNbZrTi)_1/6_O_3_ and e) Pb(ZrTi)_1/2_O_3_. f) Comparison of EMD curves between Pb(NiWMnNbZrTi)_1/6_O_3_ and Pb(ZrTi)_1/2_O_3_. g) Stress distribution diagram of Pb(NiWMnNbZrTi)_1/6_O_3_ system under the fluid field (the left and right ruler represents the stress distribution of fluid and particle, respectively). Surface charge map of the h) Pb(NiWMnNbZrTi)_1/6_O_3_ and i) Pb(ZrTi)_1/2_O_3_ particle at steady state.

To gain an understanding of the active structure of Pb(NiWMnNbZrTi)_1/6_O_3_, *in situ* Raman spectra were performed in 0.1 M KOH at various ORR potentials (−0.2–−0.6 V *vs*. SCE). As seen in Figure 4a, the *B*‐site metal‐O bond for all metal species in Pb(NiWMnNbZrTi)_1/6_O_3_ gradually increased with the increase of the applied operation potential. This indicated that all *B*‐site metal species possibly synergistically catalyzed the O_2_ to produce H_2_O_2_. This could be due to the synergistic catalysis effect of high entropy materials although the related mechanism can be complex. Such observations have also been made for many high entropy materials in typical catalytic reactions including OER and 4 e^−^‐dominated ORR.^[25]^ It is important to note that, a change of Pb−O signal was also found in the *in situ* Raman spectra, which could be ascribed to the strong intrinsic adsorption ability of Pb to hydroxyl groups.^[26]^ Interestingly, it is worth mentioning that Pb(ZrTi)_1/2_O_3_ was first thought of as 4 e^−^ ORR electrocatalyst, however, our present investigation reveals that it can also show an intrinsic catalytic activity towards the production of H_2_O_2_. This could be verified in Figure 4b where the signals for Zr−O and Ti−O bonds increased gradually with increasing the operating voltage.

On the other hand, the TPV was applied to investigate and compare the electron transfer behavior of the Pb(NiWMnNbZrTi)_1/6_O_3_ and Pb(ZrTi)_1/2_O_3_ catalysts. TPV curves of Pb(NiWMnNbZrTi)_1/6_O_3_ and Pb(ZrTi)_1/2_O_3_ are displayed in Figure 4c. Especially, the TPV curve for Pb(NiWMnNbZrTi)_1/6_O_3_ was more oscillating as compared to that for Pb(ZrTi)_1/2_O_3_, implying the distinct electron transfer process between these two oxides. Moreover, a slower TPV signals decay tendency was observed for Pb(ZrTiMnWNiNb)_1/6_O_3_ (Figure S25) indicating the electron accumulation effect and sluggish kinetics of electron transfer.^[27]^ Thus, the formation of high entropy perovskite oxide weakens the charge transfer ability, which may block the binding of oxygen with electrons. As the 4 e^−^ ORR process requires a fast electron transfer ability and sufficient reaction of oxygen with electrons, the diminished charge depletion ability and charge transfer kinetics caused by the high entropy possibly suppressed the 4 e^−^ ORR process and facilitated the 2 e^−^ ORR process.[^9^, ^28^] To further analyze the TPV data, continuous wavelet transformation (CWT) was performed on TPV data. The three‐dimensional continuous wavelet transformation results of Pb(NiWMnNbZrTi)_1/6_O_3_ and Pb(ZrTi)_1/2_O_3_ were displayed in Figures 4d and e, in which three parameters including time, frequency, and intensity were involved. Low‐frequency denotes slow electron transportation, and high frequency represents fast electron transportation. Taking the low‐frequency 5 Hz as an example, the Pb(NiWMnNbZrTi)_1/6_O_3_ exhibits a lagging peak than that of Pb(ZrTi)_1/2_O_3_ manifesting the accumulation of electrons on the Pb(NiWMnNbZrTi)_1/6_O_3_ surface (reaction sites) rather than the transfer (inset in Figure 4c). Similarly, a comparison at higher frequencies also supported the same conclusion (Figure S26).

In order to better understand the electron transfer process on the catalyst surface, empirical mode decomposition (EMD) was also utilized to refine the meaningful TPV data. EMD is an adaptive time‐space analysis method and performs operations that partition a series into intrinsic mode functions (IMFs) without leaving the time domain. These modes contribute to various signals contained within the data. By the EMD method, the white noise is averaged out, and then the persistent part that survived from the averaging process provides real and meaningful signals (Figure 4f).^[29]^ It was found that the Pb(NiWMnNbZrTi)_1/6_O_3_ delivered a higher intensity distribution than Pb(ZrTi)_1/2_O_3_, i.e, electrons were easier to accumulate and be trapped on the surface of the Pb(NiWMnNbZrTi)_1/6_O_3_, which was consistent with the TPV and CWT results. Therefore, based on the above results, it was clear that the electron transfer on the Pb(NiWMnNbZrTi)_1/6_O_3_ surface was slower, which probably affected the binding reaction of active sites with sufficient oxygen leading to the preferrable 2 e^−^ pathway. In the case of Pb(ZrTi)_1/2_O_3_, the electrons stay on the catalytic sites for a shorter time causing more electrons to react with oxygen and allowing the whole reaction pathway to deviate from the 2 e^−^ pathway.

Furthermore, the stress, electrical, and charge distribution on the surface of Pb(NiWMnNbZrTi)_1/6_O_3_ and Pb(ZrTi)_1/2_O_3_ particle was modeled by the FEA (Figure 4g and Figure S27, S28). The results showed that both the charge (8.15×10^−8^ C) and charge density (2.03×10^−2^ C m^−2^) for Pb(NiWMnNbZrTi)_1/6_O_3_ were lower than those for Pb(ZrTi)_1/2_O_3_ (1.54×10^−7^ C, 3.69×10^−2^ C m^−2^) (Figure 4h, i). The less positive charge density on the surface of Pb(NiWMnNbZrTi)_1/6_O_3_ not only suggested the possible trap of more electrons on the surface as compared to Pb(ZrTi)_1/2_O_3_ towards catalysis but also implied its weaker binding ability with −OOH, and thus being able to easily dissociate the bond between the substrate and −OOH to produce the H_2_O_2_.

### Extended Dye Degradation Property

**Figure 5:**
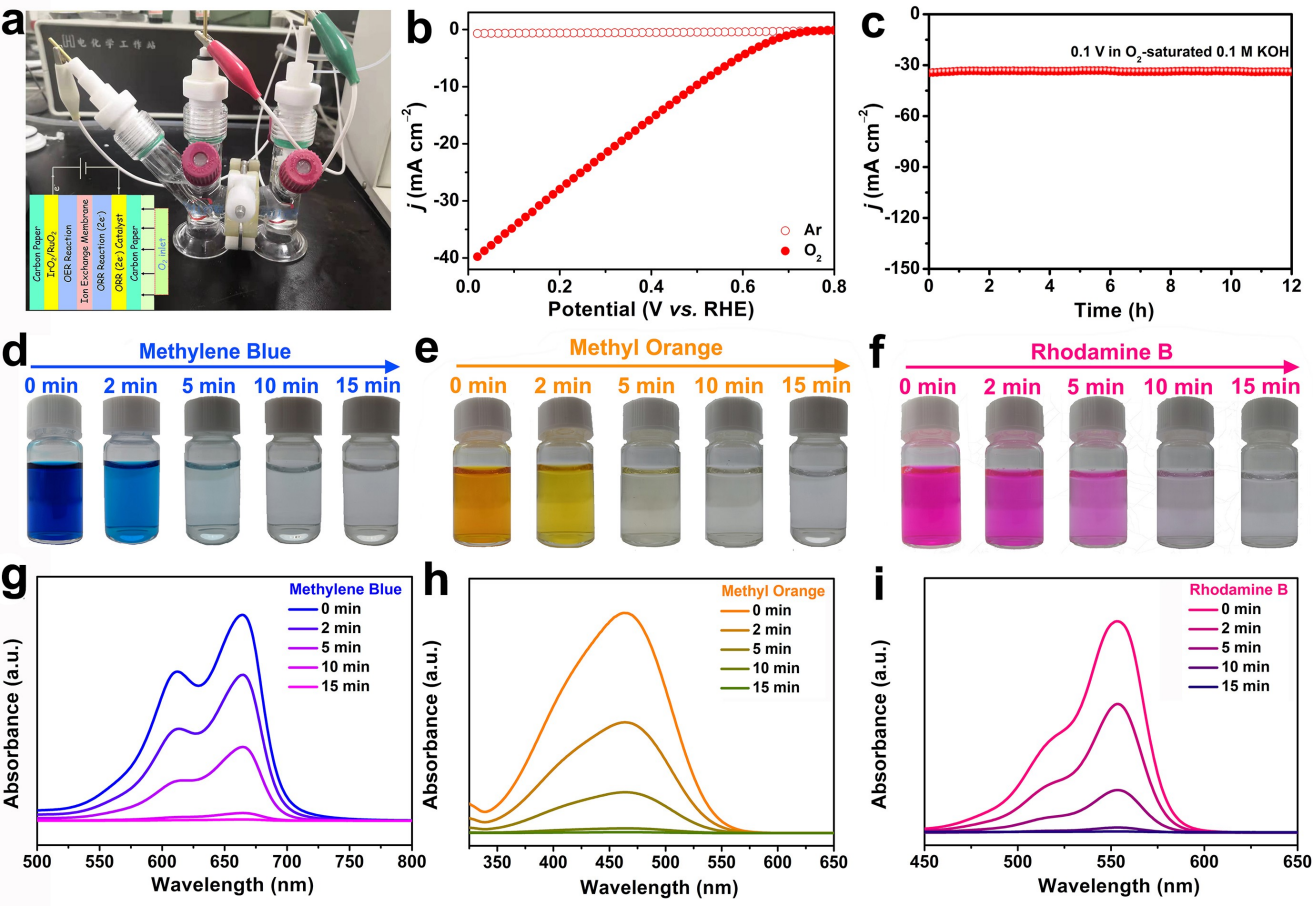
a) Digital image of H‐type electrolyzer assembled by working electrode (Pb(NiWMnNbZrTi)_1/6_O_3_ loaded onto carbon paper), counter electrode (IrO_2_ loaded onto carbon paper), and a SCE reference electrode in 0.1 M KOH. Inset in (a) shows the schematic diagram of H‐type electrolyzer. b) LSV curves of the catalyst in O_2_ and Ar‐saturated 0.1 M KOH. c) *i–t* curve of the H_2_O_2_ production in the H‐type electrolyzer. Digital images for the decolorization of d) MB, e) MO, and f) RB against the degradation time; UV/Visible spectra of the g) MB h) MO and i) RB solution at different time intervals.

Inspired by the above exciting findings, a tandem reaction was conducted by coupling ORR with dye degradation. As illustrated in Figure 5a, Pb(NiWMnNbZrTi)_1/6_O_3_ was loaded on the carbon paper, and then assembled in an H‐cell electrolyzer containing O_2_‐saturated 0.1 M KOH solution. Figure 5b shows the LSV curves obtained under the Ar and O_2_. It could be seen that the attained current density was negligible in the Ar‐saturated 0.1 M KOH, while a large current density was observed in the O_2_‐saturated 0.1 M KOH, which demonstrated a strong oxygen reduction ability of Pb(NiWMnNbZrTi)_1/6_O_3_. Subsequently, the electrolytic cell was discharged at 34 mA cm^−2^ for 30 minutes, and then an appropriate amount of electrolyte in the cathode area was taken out, acidified, and then reacted with Ce^4+^ to determine H_2_O_2_ productivity. Remarkably, in addition to the excellent activity and selectivity, the Pb(NiWMnNbZrTi)_1/6_O_3_ led to a H_2_O_2_ production rate as high as 1.1 mol g_cat_^−1^ h^−1^. Besides, the long‐term stability test result delivered only a very minor current decrease (≈2 %) even after 12 h (Figure 5c).

It is well known that the Fenton reaction between H_2_O_2_ and Fe^2+^ will generate hydroxyl radicals, and therefore, the Fenton reaction is often used in treating pollutants and wastewater. Inspired by this, we further employed Pb(NiWMnNbZrTi)_1/6_O_3_ as an electrocatalyst to generate H_2_O_2_ for the degradation of organic dyes through the Fenton reaction (details in the Experimental Section). Three organic dyes, MB, MO, and RB, were used to evaluate the degradation ability. Figure 5d–f shows the optical image of MB, MO, and RB where fading of color to decolorization was observed within the first 15 mins. Figure 5g–i displays the UV/Visible absorption spectra of MB, MO, and RB. At a fixed dye concentration (30 ppm), all three dyes exhibited a large decrease in their absorption peak within the first two minutes, and no obvious absorption was visible at 15 min. Moreover, the rate of dye degradation for the investigated dyes was much faster than the previous literature reported ORR catalysts (Table S7). The presented results demonstrate that Pb(NiWMnNbZrTi)_1/6_O_3_ is not only an excellent catalyst for the large‐scale production of H_2_O_2_ but also can spontaneously be applied to effectively degrade a variety of pollutants in wastewater by the Fenton reaction.

## Conclusion

In summary, a novel high‐entropy perovskite oxide ceramic (Pb(NiWMnNbZrTi)_1/6_O_3_) was synthesized on a large scale by a thermal‐triggered solid solution method and was demonstrated as an efficient electrocatalyst for the electrochemical reduction of O_2_ to H_2_O_2_. The as‐synthesized Pb(NiWMnNbZrTi)_1/6_O_3_ was found to be a selective catalyst for the electrosynthesis of H_2_O_2_ in alkaline solution producing over 91 % of selectivity across a wide potential range from 0.1 to 0.7 V and was remarkably stable without any loss of current even after 12 h duration at 0.4 V. In addition, the entropy‐enhanced Pb(NiWMnNbZrTi)_1/6_O_3_ was directly compared with the low‐entropy Pb(ZrTi)_1/2_O_3_ that showed a significant difference in the performance of ORR. Further *in situ* and *ex situ* investigations combined with computational analysis unveiled that the entropy increase not only induced a polymorphic phase transition accompanied with a release of lattice strain but also reduced electron migration capability on the surface, thus improving structural stability as well as enhancing the catalytic activity for the selective production of H_2_O_2_. Furthermore, the robust H_2_O_2_ production enabled by Pb(NiWMnNbZrTi)_1/6_O_3_ electrode ensured the effective electro‐Fenton process and efficient degradation of several organic pollutants including MB, MO, and RB, showing enormous potential for this electrode for on‐site water treatment applications. Finally, the integration of perovskite with high entropy as well as the related findings for the application of ORR to produce H_2_O_2_ not only establishes the representative Pb(NiWMnNbZrTi)_1/6_O_3_ as the new benchmark 2 e^−^ ORR electrocatalysts in alkaline solution but also provides new mechanistic insights and opens a new design avenue for cost‐effective, stable and efficient earth‐abundant transition metal compound electrocatalysts.

## Conflict of interest

The authors declare no conflict of interest.

1

## Supporting information

As a service to our authors and readers, this journal provides supporting information supplied by the authors. Such materials are peer reviewed and may be re‐organized for online delivery, but are not copy‐edited or typeset. Technical support issues arising from supporting information (other than missing files) should be addressed to the authors.

Supporting Information

## Data Availability

The data that support the findings of this study are available from the corresponding author upon reasonable request.
