# Supplementary material for: Entropy Enhanced Perovskite Oxide Ceramic for Efficient Electrochemical Reduction of Oxygen to Hydrogen Peroxide
Source: Angew Chem Int Ed Engl. 2022 Mar 23;61(21):e202200086. doi: 10.1002/anie.202200086 (PMC9400899; doi:10.1002/anie.202200086)
Supplement: Supplementary file 1 — Supporting Information [file ANIE-61-0-s001.pdf]

Angewandte Chemie
Eine Zeitschrift der Gesellschaft Deutscher Chemiker

Supporting Information

Entropy Enhanced Perovskite Oxide Ceramic for Efficient Electrochemical Reduction of Oxygen to Hydrogen Peroxide

Z. Chen, J. Wu, Z. Chen, H. Yang, K. Zou, X. Zhao, R. Liang, X. Dong, P. W. Menezes*, Z. Kang**

Supporting Information

Entropy Enhanced Perovskite Oxide Ceramic for Efficient Electrochemical Reduction of Oxygen to Hydrogen Peroxide

Ziliang Chen, Jie Wu, Zhengran Chen, Hongyuan Yang, Kai Zou, Xiangyong Zhao, Ruihong Liang, Xianlin Dong, Prashanth W. Menezes,* and Zhenhui Kang**

Experimental Section

Materials synthesis: The ceramics reported in this work were synthesized by the high-temperature solid-phase reaction. Raw chemicals including Pb_3O_4 (99.63%), ZrO_2 (99.76%), TiO_2 (99.38%), NiO (99.95%), WO_3 (99.0%), $MnCO_3$ (99.95%), Nb_2O_5 (99.93%), Fe_2O_3 (99.0%), MoO_3 (99.5%), V_2O_5 (99.0%), and La_2O_3 (99.995%) were purchased from Sinopharm chemical reagent Co., LTD and directly used for the synthesis. During the synthesis, the raw materials were firstly mixed according to the target chemical composition, and then the wet ball mill was used to mix them evenly and baked to obtain the raw material powder. The ball milling medium was deionized water and zirconium balls. The mixed amount was according to the 1 : 4 : 1 ratio of raw materials, zirconium balls, and deionized water, and then 6 hours of planetary ball milling was performed. After the mixed raw materials were dried, the as-obtained raw material powders were compressed at a pressure of 200 MPa. The compressed powders were placed in an alumina crucible, heated at 2 °C min^{-1} to 860 °C for 2 h, cooled to room temperature with the furnace, and then mechanically crushed, finely ground, and dried to obtain a synthetic powder. Fine grinding also adopted the deionized water and zirconium balls as the medium. The referenced samples, including $PbTiO_3$, $PbZrO_3$, $Pb(ZrTi)_{1/2}O_3$, $Pb(MnNbZrTi)_{1/4}O_3$, $Pb(WNbZrTi)_{1/4}O_3$, $Pb(NiNbZrTi)_{1/4}O_3$, $Pb(NiWMnNbTi)_{1/5}O_3$, $Pb(NiWMnNbZr)_{1/5}O_3$, $Pb_{0.9}La_{0.1}(NiWMnNbZrTi)_{1/6}O_3$, and $Pb(NiWMnNbZrTiFeVMo)_{1/9}O_3$ were also synthesized under a similar procedure except using the corresponding target metal oxides and/or carbonates as raw materials and sintered in a temperature range from 800 to 860 °C.

Material Characterizations: The phase structure and abundance of as-prepared samples were obtained by powder X-ray diffraction (XRD) measurements on a D8 ADVANCE X-ray diffractometer with Cu Kα radiation (λ = 1.5406 Å). The XRD profiles were further analyzed by the Rietveld refinement program RIETAN-FP and the lattice strain based on the William-Halls method was also extracted by RIETAN-FP stimulation.[S1] During the refinement, the

linear confinement for the occupation of the multiple-metal atom was carried out on the basis of compositional analysis result. The element chemical states of samples were characterized by X-ray photoelectron spectrometry. The XPS measurements were carried out on an ESCALAB 250Xi spectrometer (Thermo Scientific, USA) equipped with a pass energy of 30 eV with a power of 100 W (10 kV and 10 mA) and a mono-chromatized AlKα X-ray (hν=1486.65 eV) source. All samples were analyzed under a pressure of less than 1.0×10^{-9} Pa. Spectra were acquired through the avantage software (Version 5.979) with a step of 0.05 eV. The metal contents in the samples were determined by using an inductively coupled plasma optical emission spectrometer (ICP-OES), (Varian 720-ES). The morphology and microstructure of samples were investigated by Field-emission scanning electron microscope (FESEM, Zeiss G500) and transmission electron microscope (TEM, FEI Talos F200X). *In-situ* Raman spectra during the ORR process were recorded by a Raman spectrometer with an excitation wavelength of 532 nm (Horiba LabRAM HR Evolution).

Electrochemical test: First, 3.6 mg of as-prepared electrocatalyst and 0.4 mg of Ketjen Black are mixed and dispersed in a mixture solution containing 600 μL H_2O, 300 μL ethanol, and 100 μL 0.5 wt.% Nafion solution. In order to obtain a uniform ink, the mixed solution was sonicated in an ice water bath for 30 min. Then 6.28 μL of the ultrasonic solution was dripped on the glassy carbon of the rotating ring disk electrode (RRDE) with an area of 0.1256 cm^2. An electrochemical workstation (760E, CHI) was used to evaluate the electrochemical performance of the catalysts. A standard three-electrode system was used to evaluate the performance of the catalyst, where the RRDE loaded with catalysts, saturated calomel electrode, and graphite rod are served as the working electrode, reference electrode, and counter electrode, respectively. The electron transfer number (n) and H_2O_2 selectivity (H_2O_2%) are calculated by the followings,

$$n = 4\frac{I_D}{I_D + I_R/N}$$

$$H_2O_2(\%) = 200\frac{I_R/N}{I_D + I_R/N}$$

where I_R represents the ring current, I_D represents the absolute value of disk current and the collection efficiency (N) of the RRDE is equal to 0.37.

H-cell electrolyzer and organic dyes degradation: The H_2O_2 yield of the catalyst in 0.1 M KOH was determined using an H-cell electrolyzer, in which the catalyst-loaded carbon paper was used as the working electrode, and the commercial IrO_2 loaded carbon paper was used as the counter electrode, and the mass loading of the catalysts both are 0.5 mg cm^{-2}. The catholyte containing HO_2^- is taken out and acidified by adding sulfuric acid. The H_2O_2 yield determination was quantified through the reaction between cerium sulfate ($CeSO_4$) and H_2O_2: $2Ce^{4+} + H_2O_2 \rightarrow 2Ce^{3+} + 2H^+ + O_2$. The relationship between the concentration of Ce^{4+} and absorbance was calibrated by ultraviolet-visible spectrum (UV-vis). Three organic dyes with a concentration of 30 ppm were prepared, namely rhodamine B, methyl orange, and methylene blue. Firstly, 5 mL of catholyte is collected from the cathode area after discharging the electrolytic cell at a current density of 35 mA cm^{-2} for 30 min. Secondly, 1 mL 1 M H_2SO_4 and 1×10^{-3} Fe^{2+} were added into the obtained 5 mL catholyte to acidizing the solution. Finally, the above-acidified solution containing Fe^{2+} was mixed with 10 mL of dyes solution, shaking continuously to ensure a sufficient reaction (Fenton reaction). Dyes are degraded by hydroxyl radicals (OH·) generated by Fenton reaction: $2Fe^{2+} + H_2O_2 \rightarrow 2Fe^{3+} + OH^- + OH\cdot$. The concentration of the dyes at different moments is determined using a UV-Vis, and the absorption peaks of the three dyes are located at 664 nm (methylene blue), 463 nm (methyl orange), and 553 nm (rhodamine b), respectively.

TPV principle: A stimulus response method, called transient phototelepressure (TPV) measurement, was performed on a homemade measurement system with a platinum net covering a power sample (1 cm * 1 cm) as the working electrode and a Pt line as the counter

electrode sample at room temperature from a third harmonic Nd: YAG laser (Polaris II, New) Wave Research, Inc.) laser radiation pulse (λ = 355 nm, pulse width 5 ns) excited TPV signal is first amplified, and then the oscilloscope records the photocurrent is the ratio of the photovoltage to the internal resistance of the test system.

Finite Element Analysis: The finite element analysis (FEA) can simulate various physical and chemical properties/reaction processes at mesoscopic scale, thus being widely employed in different research fields, including mechanics, fluids, electromagnetics, optics, and electrochemistry [S2]. More intriguingly, the FEA simulation can simplify the complexity of the reaction models to reflect the general trend, especially at the dynamic proceeding where vague and changeable intermediates are involved. Consequently, during the electrochemistry, the application of FEA can shed more lights on the reactions at the interface between the electrode and electrolyte, which are typically neglected before. This is because the dominant theoretical calculation in this area is based on the first-principle method (e.g., density functional theory, DFT), which is confined at modeling atomic configurations (revolving around the specific active atomic sites) where the energetics of elementary reaction processes are calculated. Furthermore, the influences resulting from the externally applied electric field and structural evolution of electrodes at longer timescale which are beyond the DFT calculation regime can also be captured by FEA [S3]. In the specific area of electrocatalysis, the FEA was more extensively utilized to simulate the properties, such as electric field distribution, direction, and intensity, of the surface of the electrode, which then can estimate the surrounding concentration of the reactants or intermediate ions, thereby reflecting the catalysis kinetics [S2f, 4]. Similarly, in the conventional electrochemical cell, the pH value and potential in the immediate vicinity of the electrode can also be assessed [S5]. Based on these, now it can be observed that FEA methods emerged in different electrolysis, including hydrogen evolution reaction [S6], oxygen evolution reaction [S4], carbon dioxide reaction [S2f], providing deeper insights into the catalysis mechanisms. The stress, electrical field, and charge distribution for

the $Pb(ZrTi)_{1/2}O_3$ and $Pb(NiWMnNbZrTi)_{1/6}O_3$ particle during ORR were modeled by the COMSOL Multiphysics® 5.5 software which was based on the Finite Element Analysis.[S7] Due to the high calculation and complexity of the actual three-dimensional model, the actual three-dimensional model is reasonably simplified to a two-dimensional model under the premise of fully ensuring that it is consistent with the actual physical process.[S8] The particle size was set as 2 μm, and the length and width of the electrode substrate was set as 50 and 5 μm, respectively (Figure S29). After the geometric model was successfully created, the material property was assigned according to the actual experimental settings. Specifically, the piezoelectricity constant and relative permittivity for $Pb(NiWMnNbZrTi)_{1/6}O_3$ were set as the 0 pC N^{-1} and 1300, respectively, while those for $Pb(ZrTi)_{1/2}O_3$ were set as the 520 pC N^{-1} (isotropy) and 2400, respectively. The actual physical process involved the three-field coupling of fluid field, electrostatic field and solid mechanic field. In the experiment, the material was in a high-speed flowing fluid, so a fluid field was applied to simulate the actual experimental process. The specific settings of the fluid field were shown in Table S8. The electrostatic field was a description of the electrostatic response of the model. Because the actual electrochemical reaction process was quite complicated, a change in the surface potential of 0.7 V *vs*. RHE was applied to the powder to simplify the expression. The reason why the potential was selected is due to the thermodynamically theoretical voltage of producing peroxide hydrogen. The mechanical response of structural materials was simulated by the solid mechanic field.

The speed f of the sample and electrode in the electrolyte solution was set to 1600 r min^{-1}, the radius of rotation r is set to 5 cm, and the steady-state average flow velocity U (8.38 m s^{-1}) at the inlet could be solved according to the following formula:

$$U = 2\pi \cdot f \cdot r \qquad (1)$$

The mesh division was automatically performed through the software, and the division result is shown in Figure S30. The transient study was also added, and the time step and the calculation time was set to 0.05 and 4 s, respectively.

FIGURES

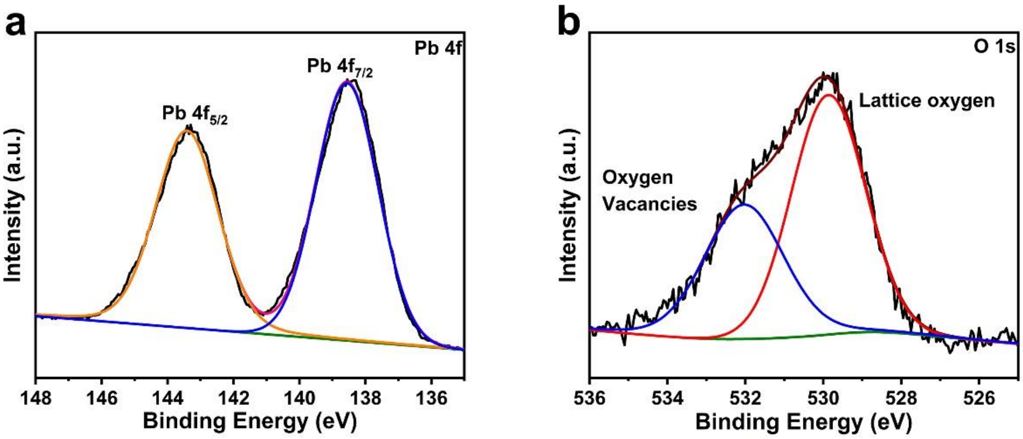

Figure S1. The high-resolution XPS of (a) Pb 4f and (b) O 1s in the $Pb(NiWMnNbZrTi)_{1/6}O_3$.

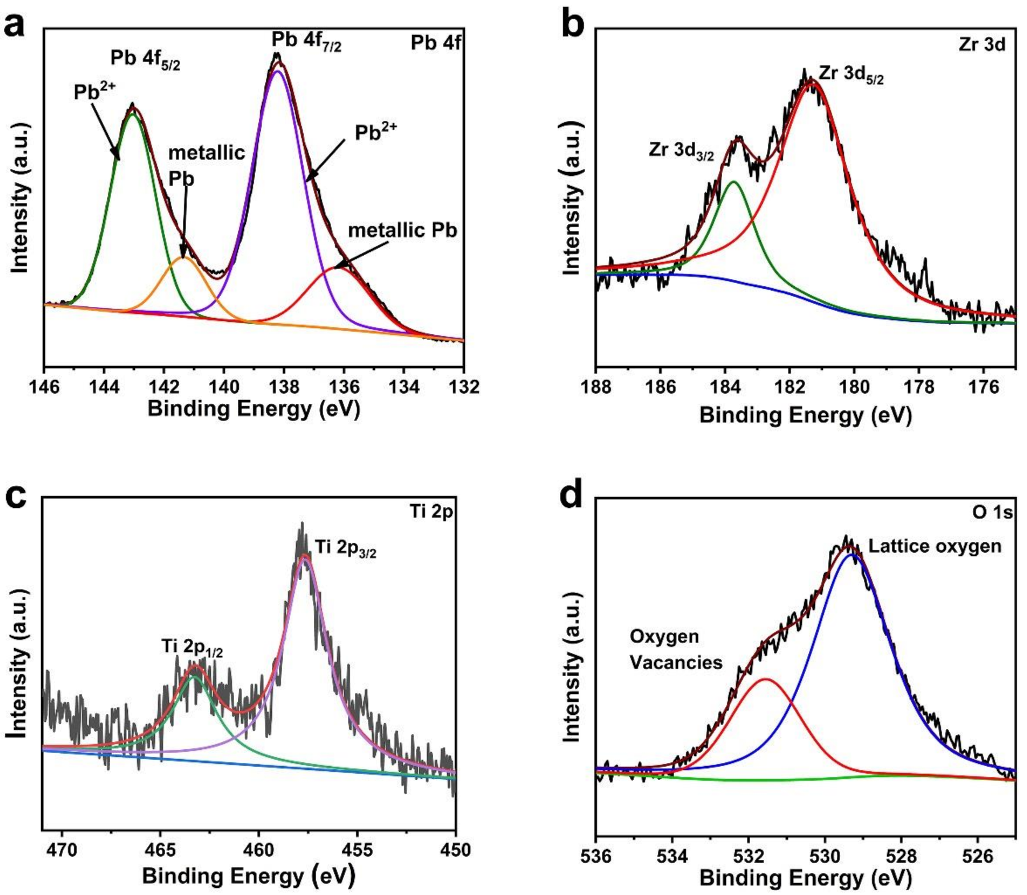

Figure S2. The high-resolution XPS of (a) Pb 4f, (b) Zr 3d, (c) Ti 2p and (d) O 1s in the $Pb(ZrTi)_{1/2}O_3$.

Description of XPS spectra of $Pb(ZrTi)_{1/2}O_3$

The high-resolution XPS spectra of $Pb(ZrTi)_{1/2}O_3$ were shown in Figure S2. In Pb 4f XPS, the metallic Pb signals were found at 141.4 eV and 136.2 eV, while the Pb^{2+} peaks located at both 143.0 and 138.2 eV possessed 0.3 eV negative shift than those of Pb in $Pb(NiWMnNbZrTi)_{1/2}O_3$ (Figure S2a). Likewise, the Zr-O bond in $Pb(ZrTi)_{1/2}O_3$ observed at 183.7 (Zr $3d_{3/2}$) and 181.2 eV (Zr $3d_{5/2}$) were about 0.6 eV lower than those of Zr 3d in $Pb(NiWMnNbZrTi)_{1/6}O_3$ (Figure S2b). In contrast, the Ti-O bonds showed in Figure S2c shifted towards higher binding energy when entropy was increased. Moreover, the high-resolution O 1s XPS spectra in $Pb(ZrTi)_{1/2}O_3$ also showed a negative shift than that of $Pb(NiWMnNbZrTi)_{1/6}O_3$ (Figure S2d).

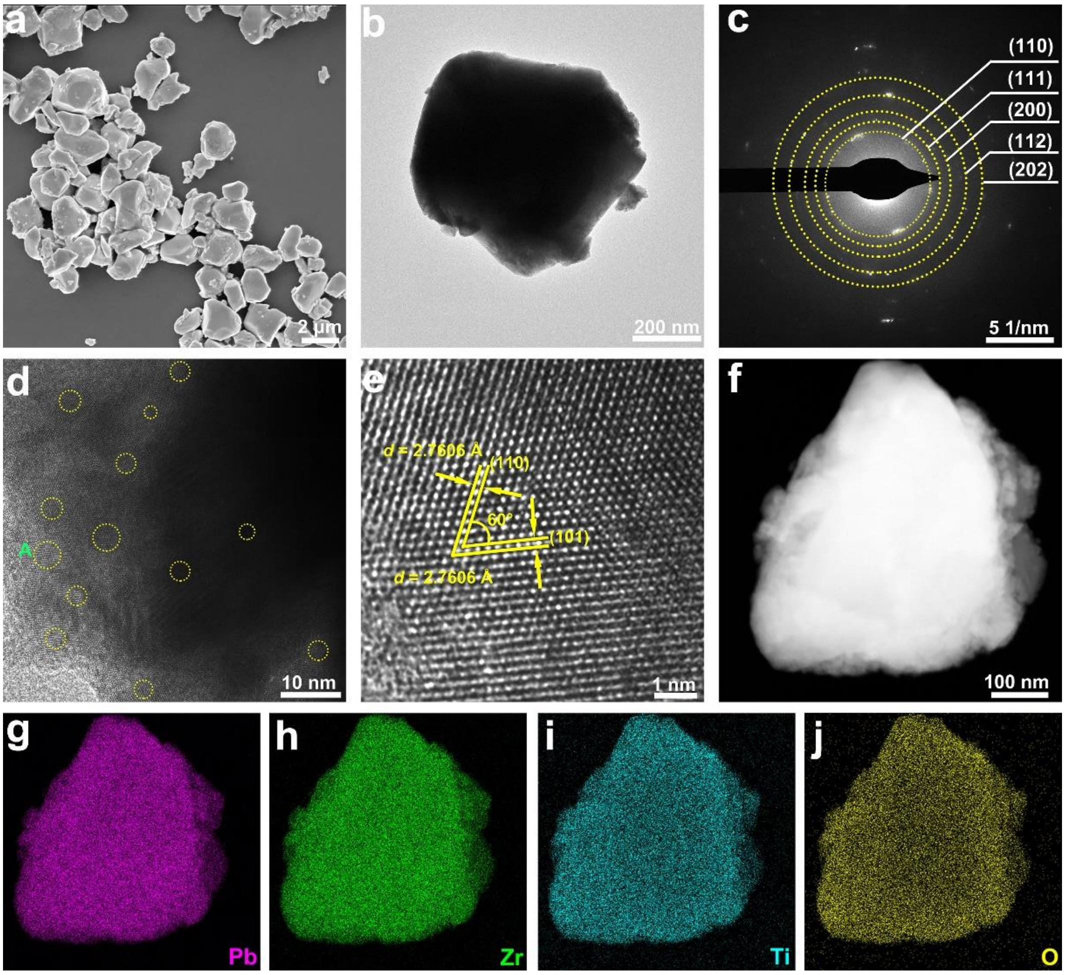

Figure S3. (a) FESEM image of Pb(ZrTi)$_{1/2}$O$_3$; (b) TEM image of Pb(ZrTi)$_{1/2}$O$_3$ and (c) corresponding SAED pattern, (d) high-magnified TEM image and (e) high-resolution TEM images; (f) HAADF image of representative Pb(ZrTi)$_{1/2}$O$_3$ particle and corresponding elemental mapping of (g) Pb, (h) Zr, (i) Ti and (j) O.

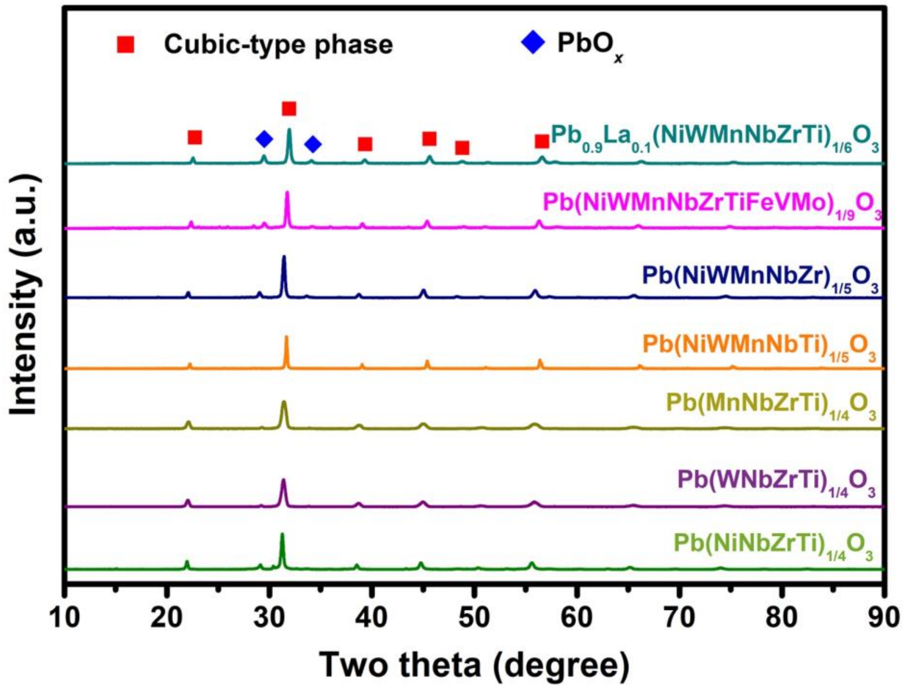

Figure S4. The XRD patterns of Pb(NiNbZrTi)$_{1/4}$O$_3$, Pb(WnbZrTi)$_{1/4}$O$_3$, Pb(MnNbZrTi)$_{1/4}$O$_3$, Pb(NiWMnNbTi)$_{1/5}$O$_3$, Pb(NiWMnNbZr)$_{1/5}$O$_3$, Pb$_{0.9}$La$_{0.1}$(NiWMnNbZrTi)$_{1/6}$O$_3$ and Pb(NiWMnNbZrTiFeVMo)$_{1/9}$O$_3$. Note that a second phase PbO$_x$ was found in some as-prepared samples. Because the diffraction peaks signal of such secondary phase was much smaller than those of cubic-type phase, we assumed that it played a little effect on ORR activity.

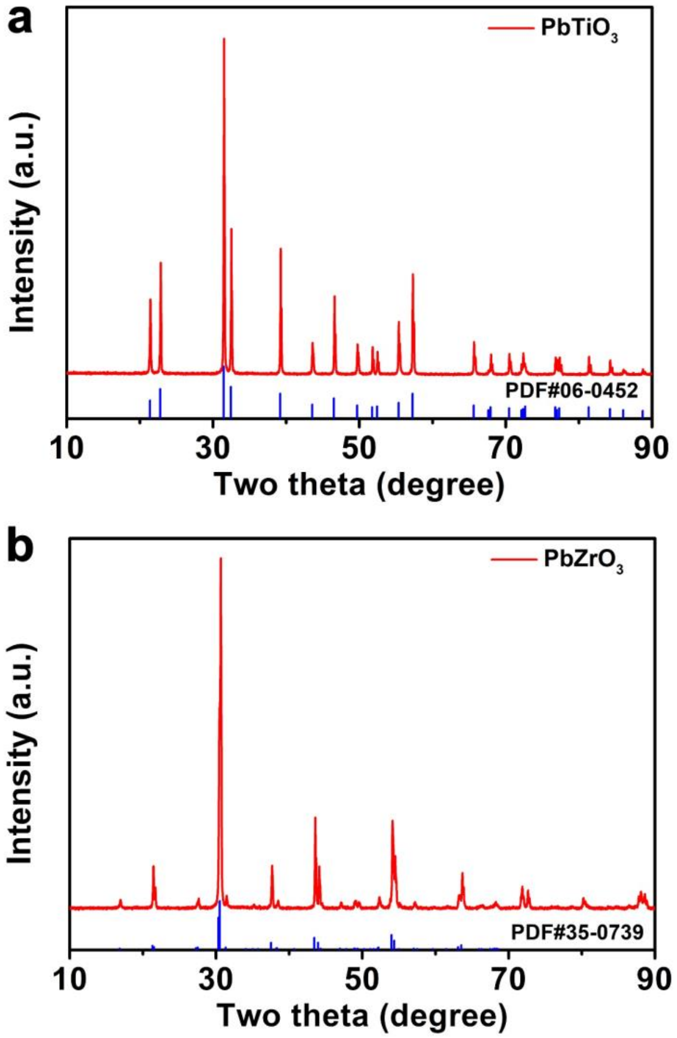

Figure S5. XRD patterns of (a) $PbTiO_3$ and (b) $PbZrO_3$ compounds.

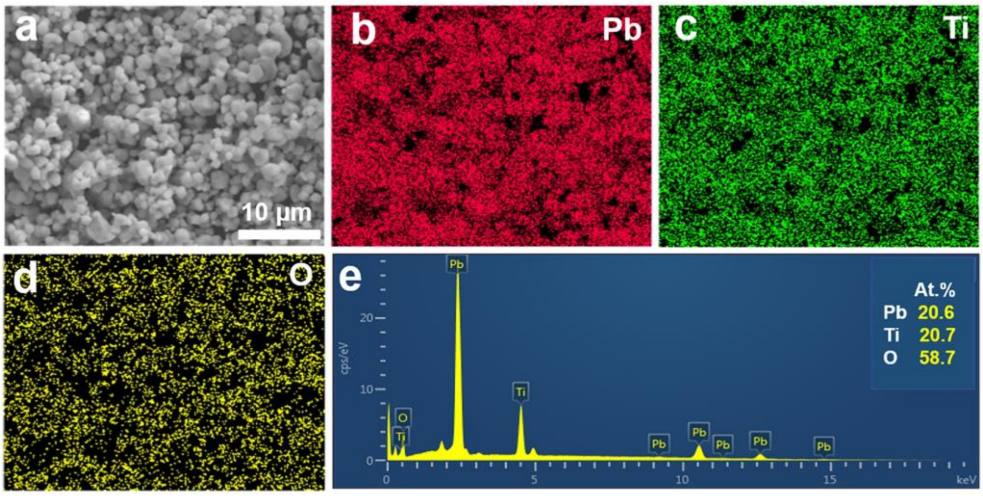

Figure S6. (a) The SEM image of $PbTiO_3$ and corresponding elemental mapping of (b) Pb, (c) Ti, (d) O, and (e) the EDX results.

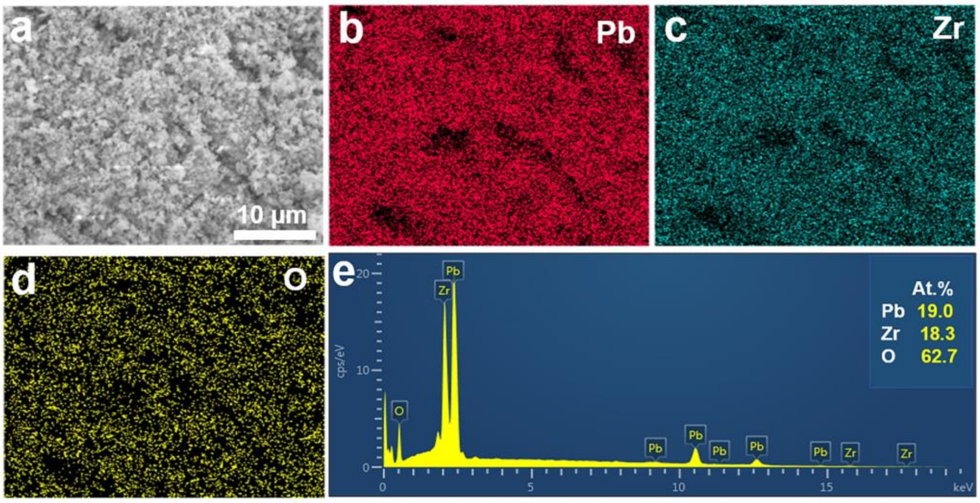

Figure S7. (a) The SEM image of $PbZrO_3$ and corresponding elemental mapping of (b) Pb, (c) Zr, (d) O, and (e) the EDX results.

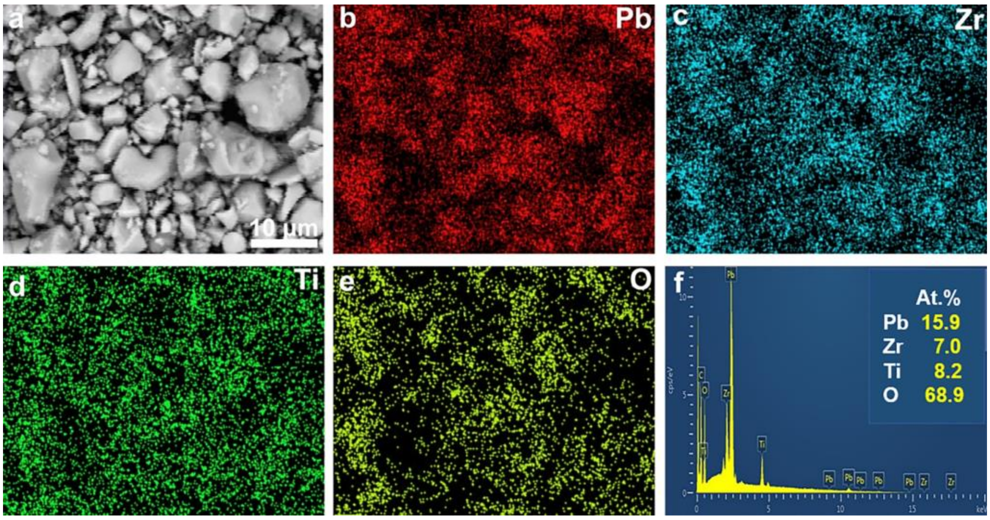

Figure S8. (a) The SEM image of $Pb(ZrTi)_{1/2}O_3$ and corresponding elemental mapping of (b) Pb, (c) Zr, (d) Ti, and (e) O, and (f) the EDX results.

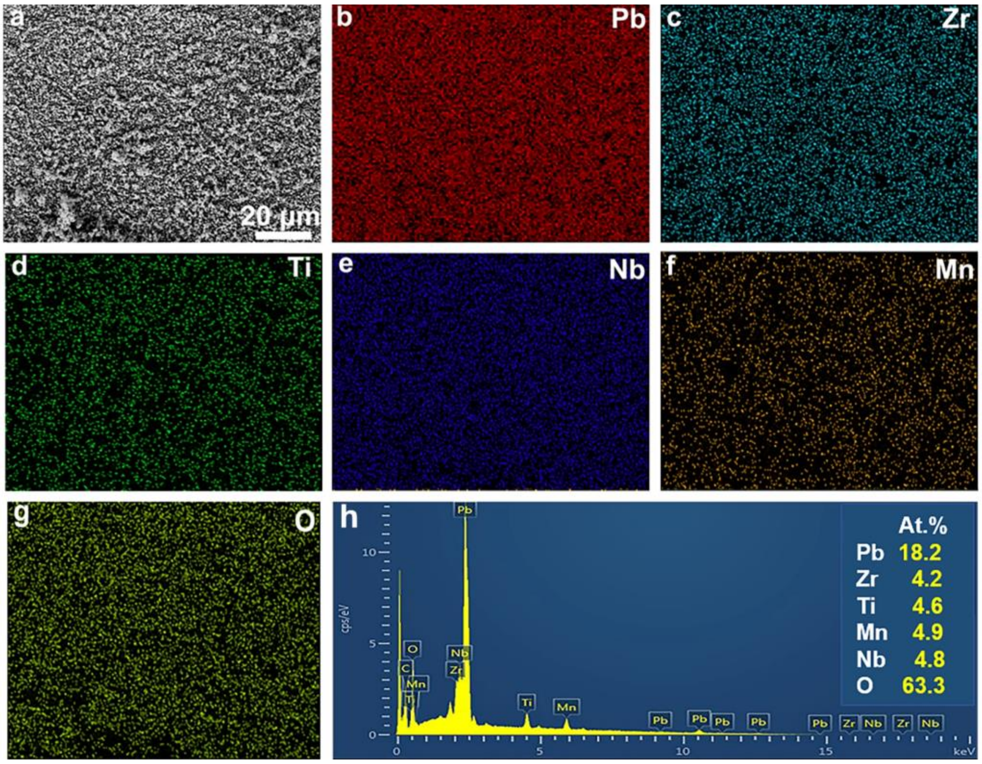

Figure S9. (a) The SEM image of Pb(MnNbZrTi)$_{1/4}$O$_3$ and corresponding elemental mapping of (b) Pb, (c) Zr, (d) Ti, (e) Nb, (f) Mn, and (g) O, and (h) the EDX results.

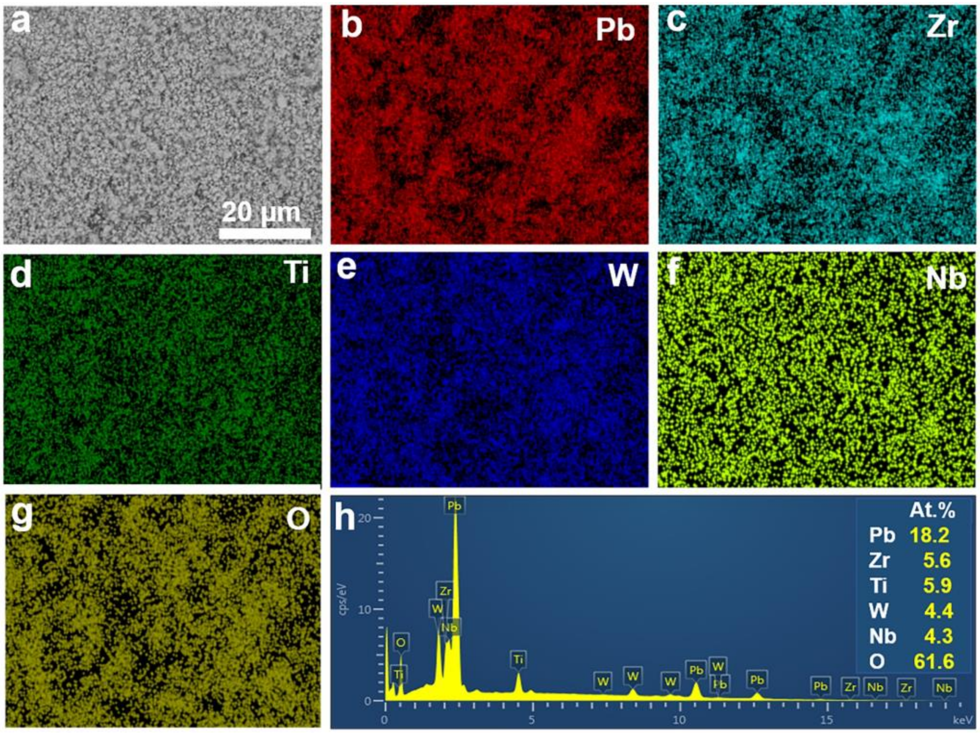

Figure S10. (a) The SEM image of $Pb(WNbZrTi)_{1/4}O_3$ and corresponding elemental mapping of (b) Pb, (c) Zr, (d) Ti, (e) W, (f) Nb, and (g) O, and (h) the EDX results.

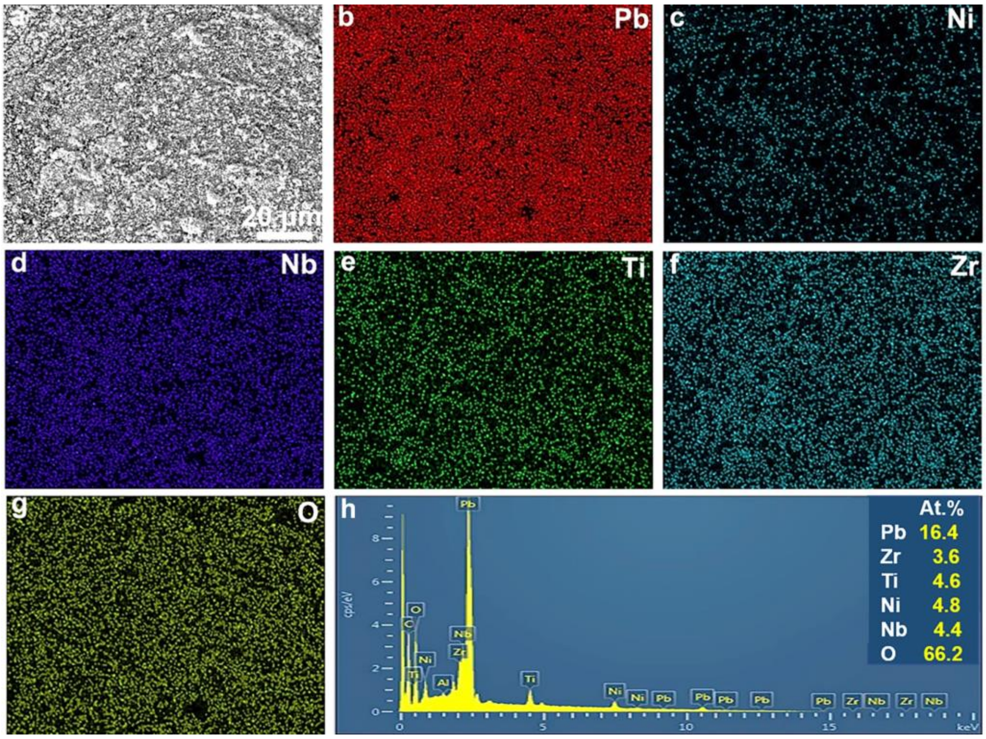

Figure S11. (a) The SEM image of $Pb(NiNbZrTi)_{1/4}O_3$ and corresponding elemental mapping of (b) Pb, (c) Ni, (d) Nb, (e) Ti, (f) Zr, and (g) O, and (h) the EDX results.

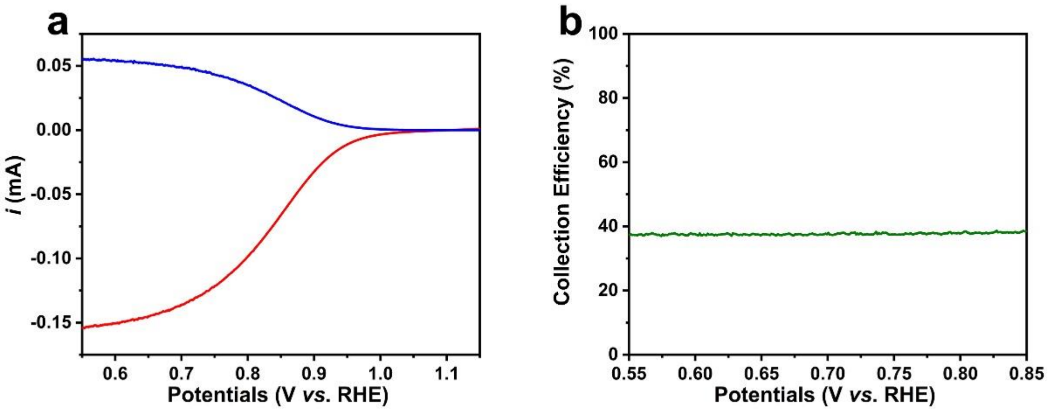

Figure S12. Calibration of the collection efficiency of the bare ring disk electrode (RRDE) in Ar-saturated 1 M KNO_3 dissolved with 2 mM $K_3[Fe(CN)_6]$. (a) RRDE voltammograms recorded by performing LSV on disk from 0.55 V to 1.15 V at 10 mV s^{-1} at 1600 rpm while holding the ring at 0.64 V *vs.* RHE. (b) The corresponding collection efficiency of RRDE voltammograms as a function of the potential.

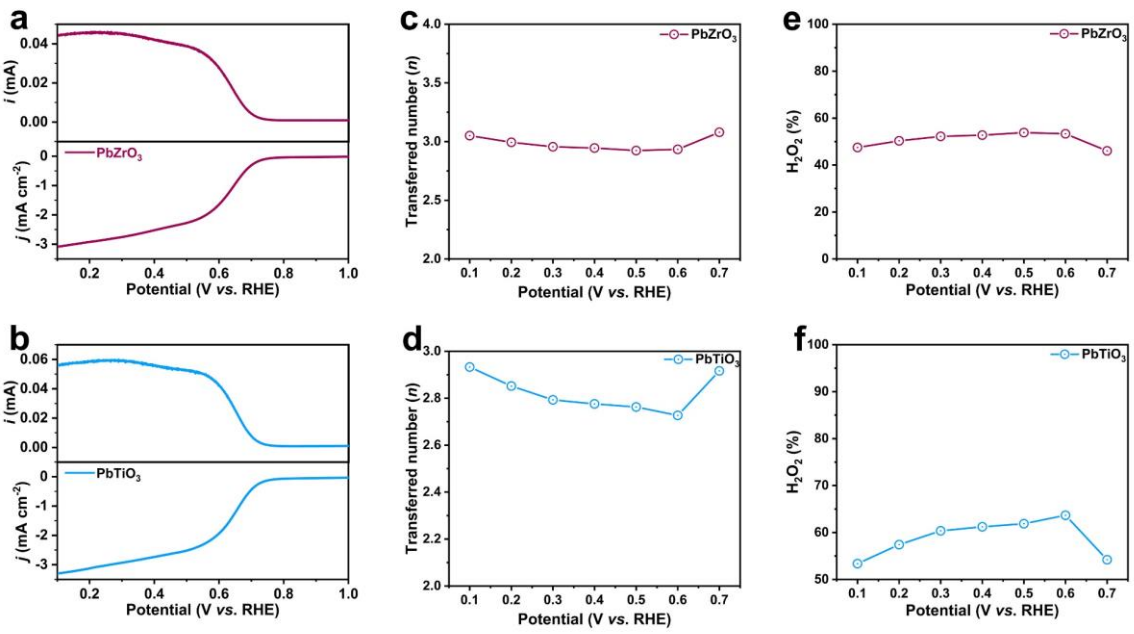

Figure S13. LSV curves of (a) $PbZrO_3$ and (b) $PbTiO_3$ recorded at 1600 rpm and 10 mV s^{-1}, together with the corresponding H_2O_2 current on the ring electrode; (c, d) Calculated electron transfer number and (e, f) selectivity of H_2O_2 within the potential sweep.

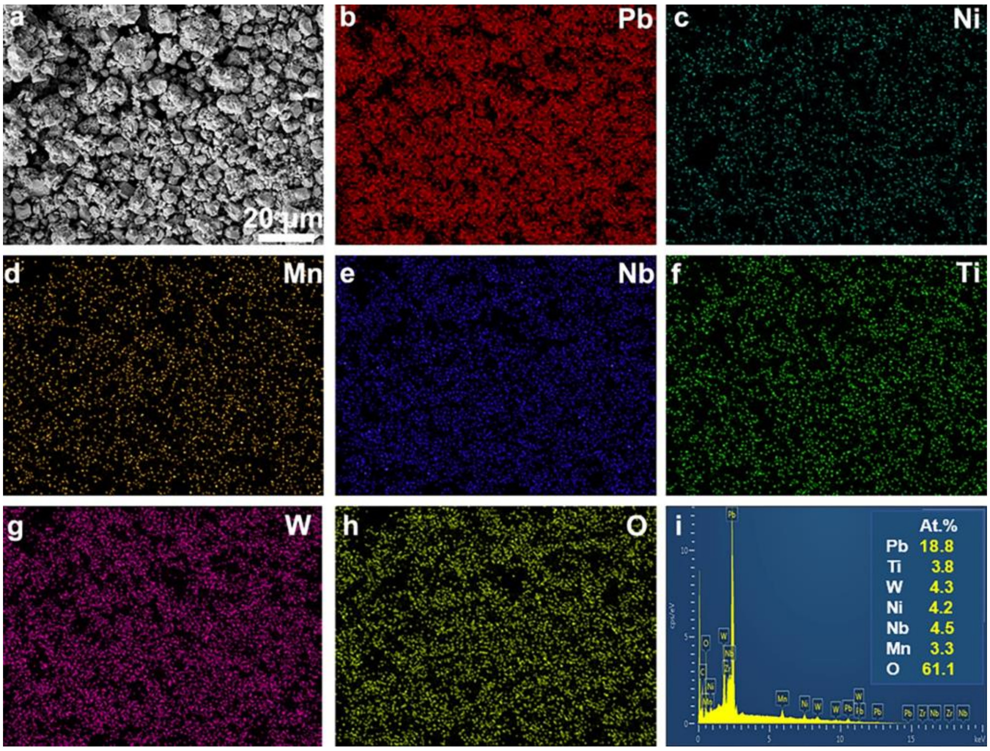

Figure S14. (a) The SEM image of $Pb(NiWMnNbTi)_{1/5}O_3$ and corresponding elemental mapping of (b) Pb, (c) Ni, (d) Mn, (e) Nb, (f) Ti, (g) W and (h) O, and (i) the EDX results.

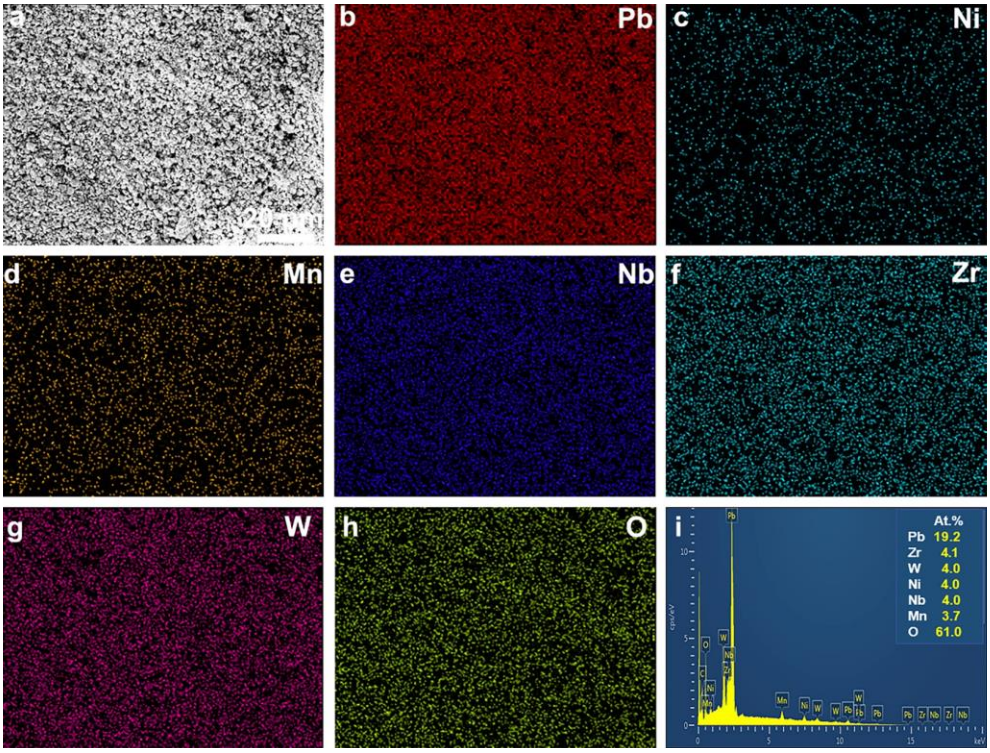

Figure S15. (a) The SEM image of $Pb(NiWMnNbZr)_{1/5}O_3$ and corresponding elemental mapping of (b) Pb, (c) Ni, (d) Mn, (e) Nb, (f) Zr, (g) W, and (h) O, and (i) the EDX results.

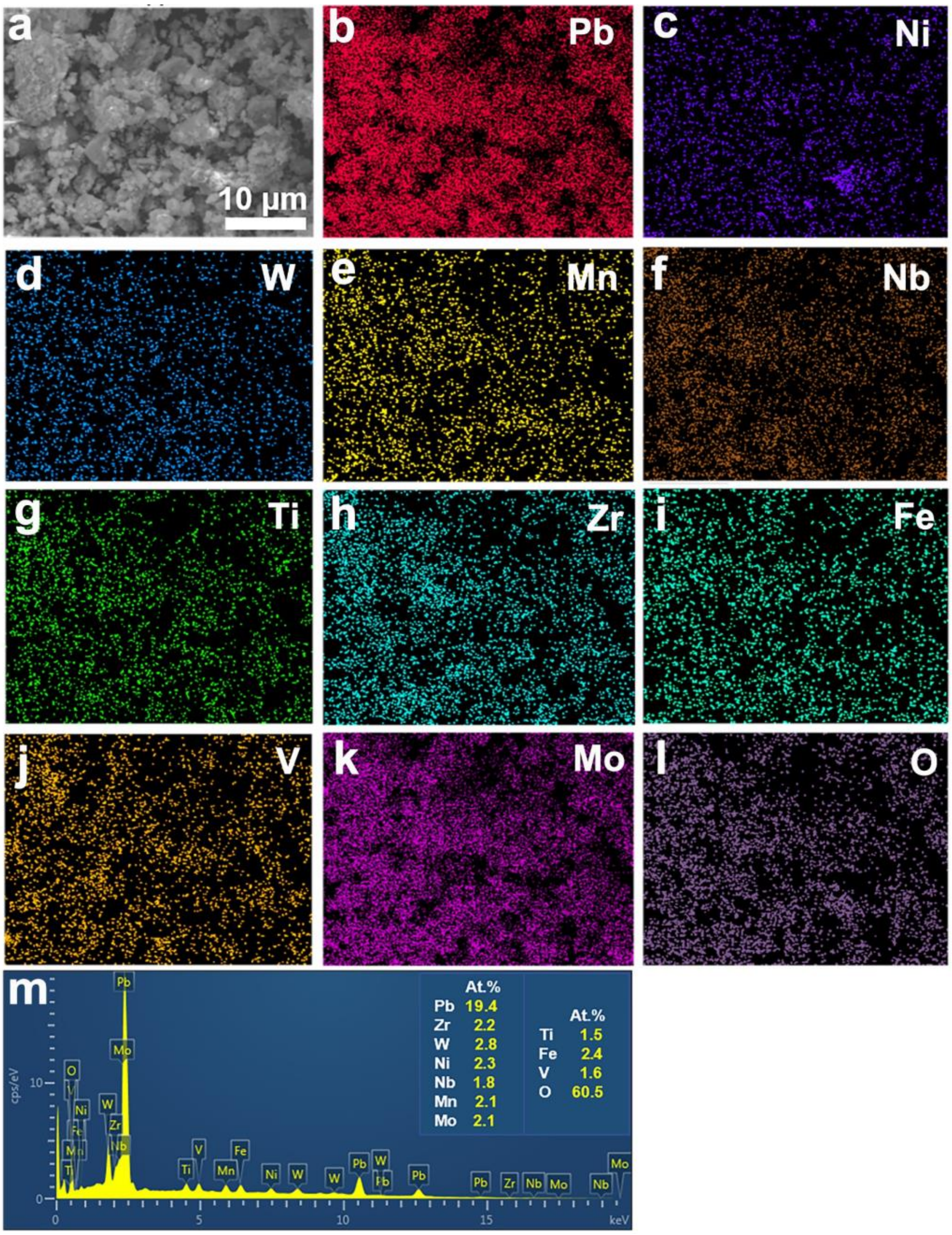

Figure S16. (a) The SEM image of $Pb(NiWMnNbTiZrFeVMo)_{1/9}O_3$, and corresponding elemental mapping of (b) Pb, (c) Ni, (d) W, (e) Mn, (f) Nb, (g) Ti, (h) Zr, (i) Fe, (j) V, (k) Mo and (l) O, and (m) the EDX results.

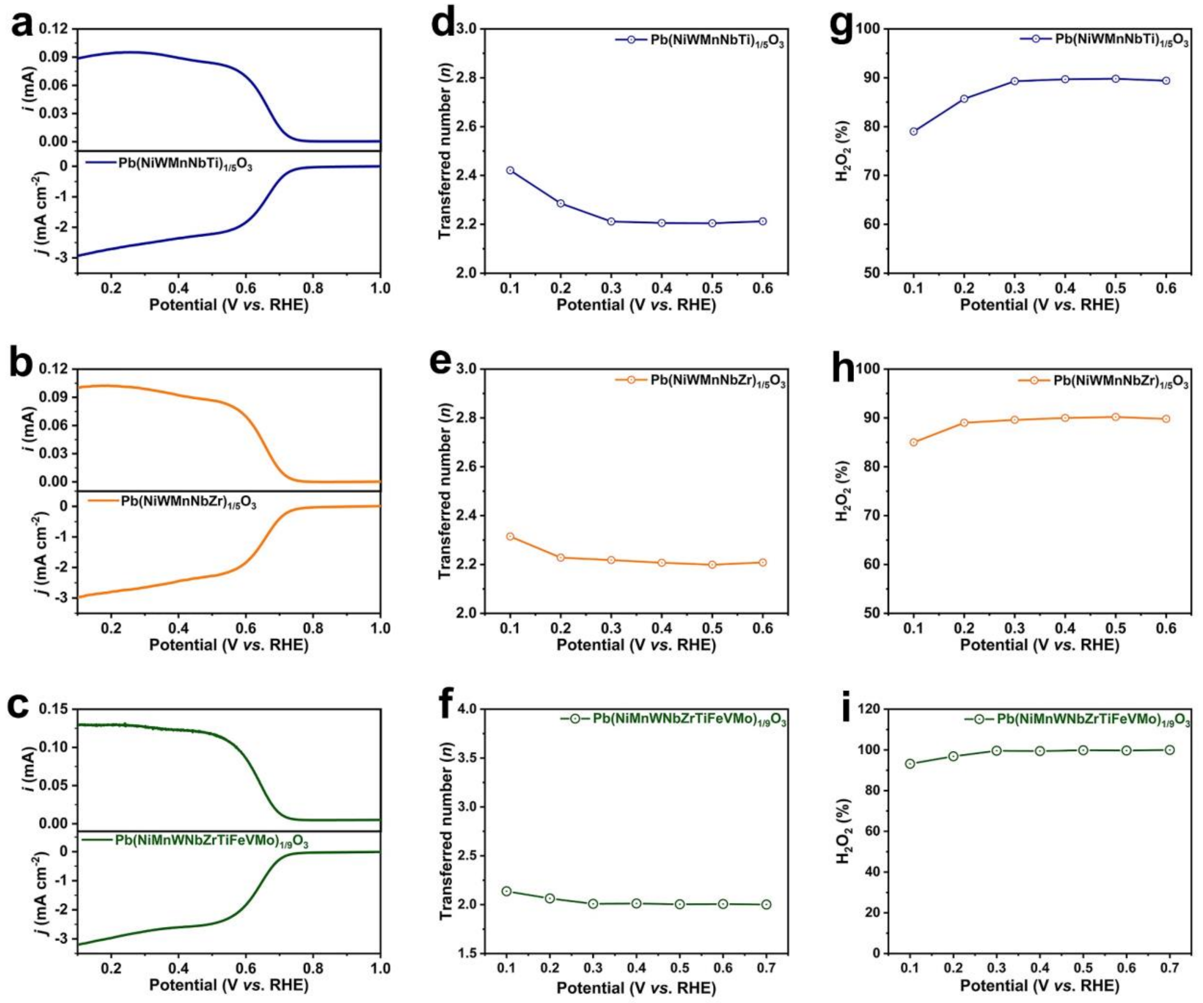

Figure S17. LSV curves of (a) Pb(NiWMnNbTi)$_{1/5}$O$_3$, (b) Pb(NiWMnNbZr)$_{1/5}$O$_3$, and (c) Pb(NiMnWNbZrTiFeVMo)$_{1/9}$O$_3$ recorded at 1600 rpm and 10 mV s^{-1}, together with the corresponding H_2O_2 current on the ring electrode; (d, e, f) Calculated electron transfer number and (g, h, i) selectivity of H_2O_2 within the potential sweep.

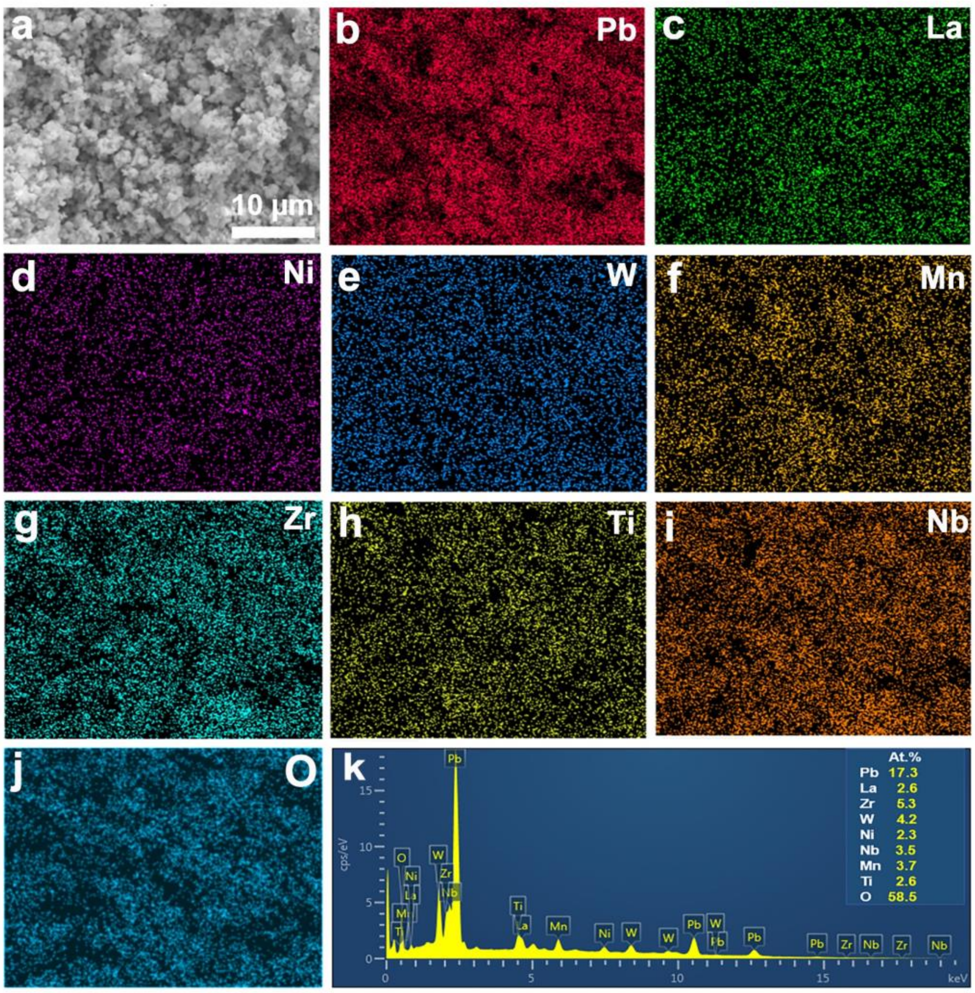

Figure S18. (a) The SEM image of $Pb_{0.9}La_{0.1}(NiWMnNbZrTi)_{1/6}O_3$ and corresponding elemental mapping of (b) Pb, (c) La, (d) Ni, (e) W, (f) Mn, (g) Zr, (h) Ti, (i) Nb and (j) O, and (j) the EDX results.

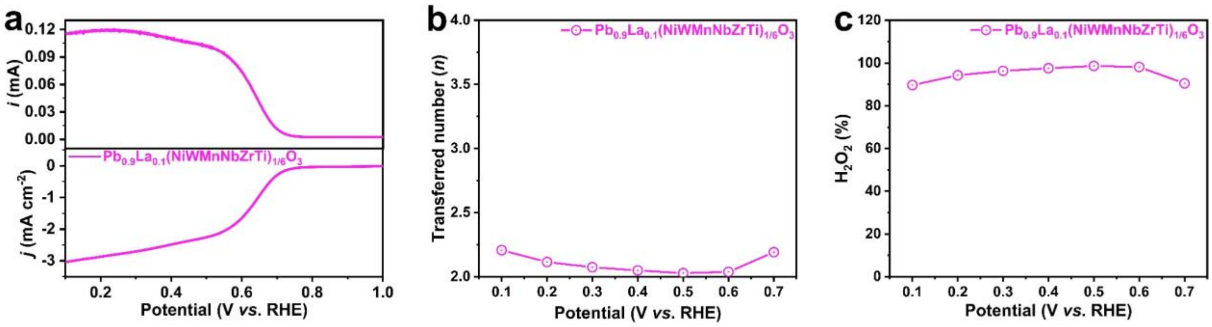

Figure S19. (a) LSV curve of $Pb_{0.9}La_{0.1}(NiWMnNbZrTi)_{1/6}O_3$ recorded at 1600 rpm and 10 mV s^{-1}, together with the corresponding H_2O_2 current on the ring electrode; (b) Calculated electron transfer number and (c) selectivity of H_2O_2 within the potential sweep.

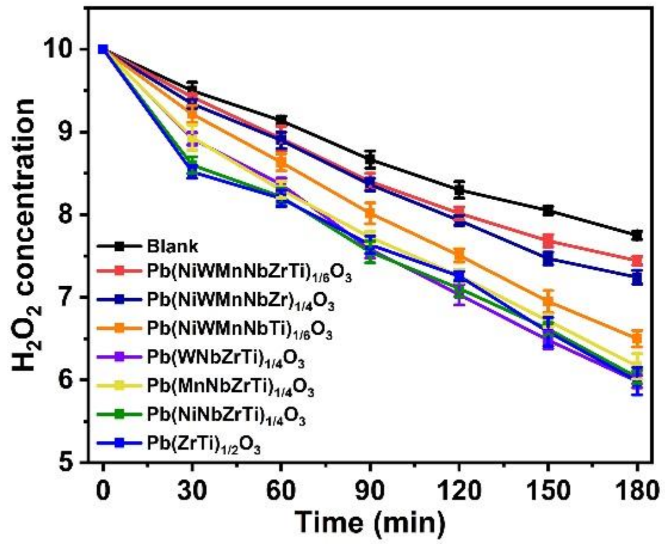

Figure S20. Peroxide disproportionation reaction (PDR) measurements. 5 mg electrocatalyst was added into 20 mL 0.1 M KOH containing 10 mM H_2O_2. For a better comparison, PDR measurement was also investigated without any electrocatalysts.

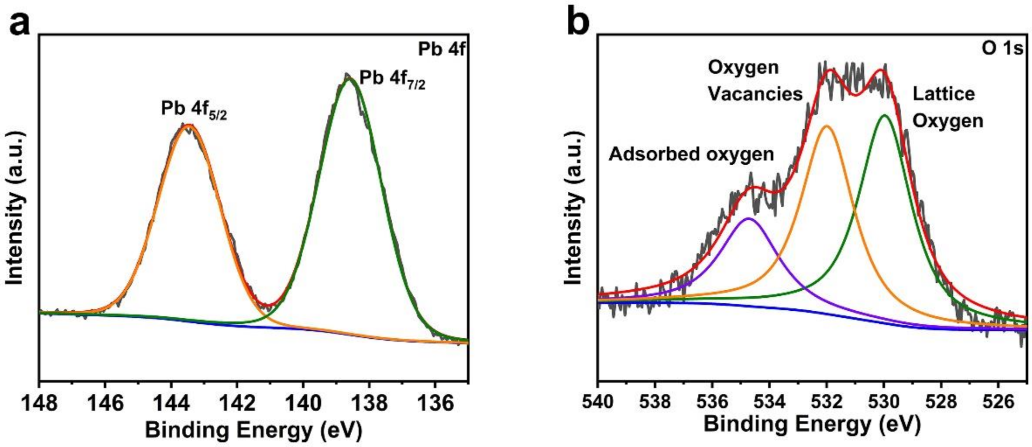

Figure S21. The high-resolution XPS of (a) Pb 4f and (b) O 1s in the Pb(NiWMnNbZrTi)$_{1/6}$O$_3$ after the chronoamperometry test.

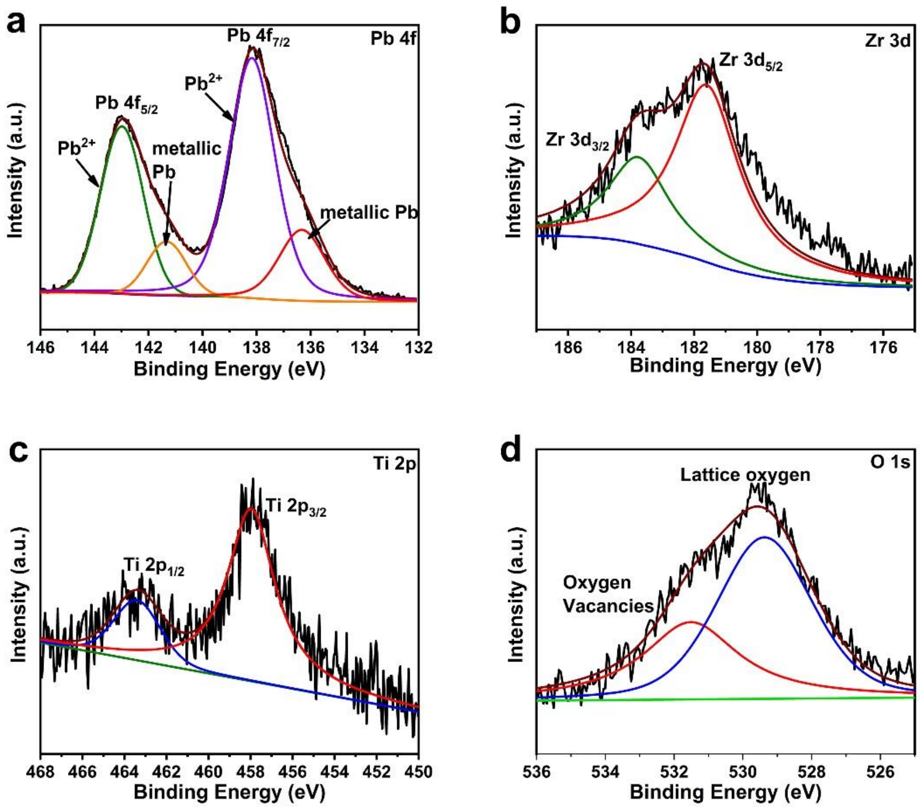

Figure S22. The high-resolution XPS of (a) Pb 4f, (b) Zr 3d, (c) Ti 2p and (d) O 1s in the $Pb(ZrTi)_{1/2}O_3$ after the chronoamperometry test**.**

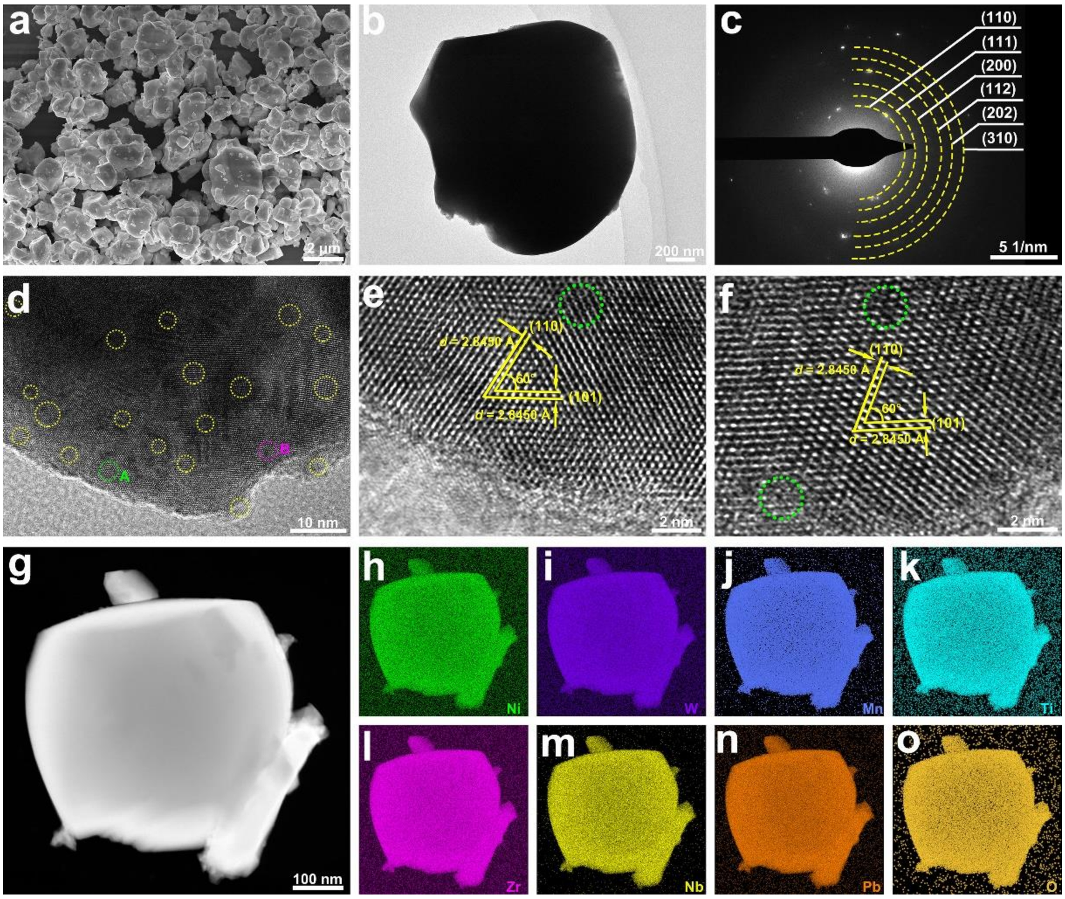

Figure S23. (a) FESEM image of $Pb(NiWMnNbZrTi)_{1/6}O_3$ after chronoamperometry test; (b) TEM image of $Pb(NiWMnNbZrTi)_{1/6}O_3$ and (c) corresponding SAED pattern, (d) high-magnified TEM image and (e, f) high-resolution TEM images; (g) HAADF image of representative $Pb(NiWMnNbZrTi)_{1/6}O_3$ particle and corresponding elemental mapping of (h) Ni, (i) W, (j) Mn, (k) Ti, (l) Zr, (m) Nb, (n) Pb, and (o) O.

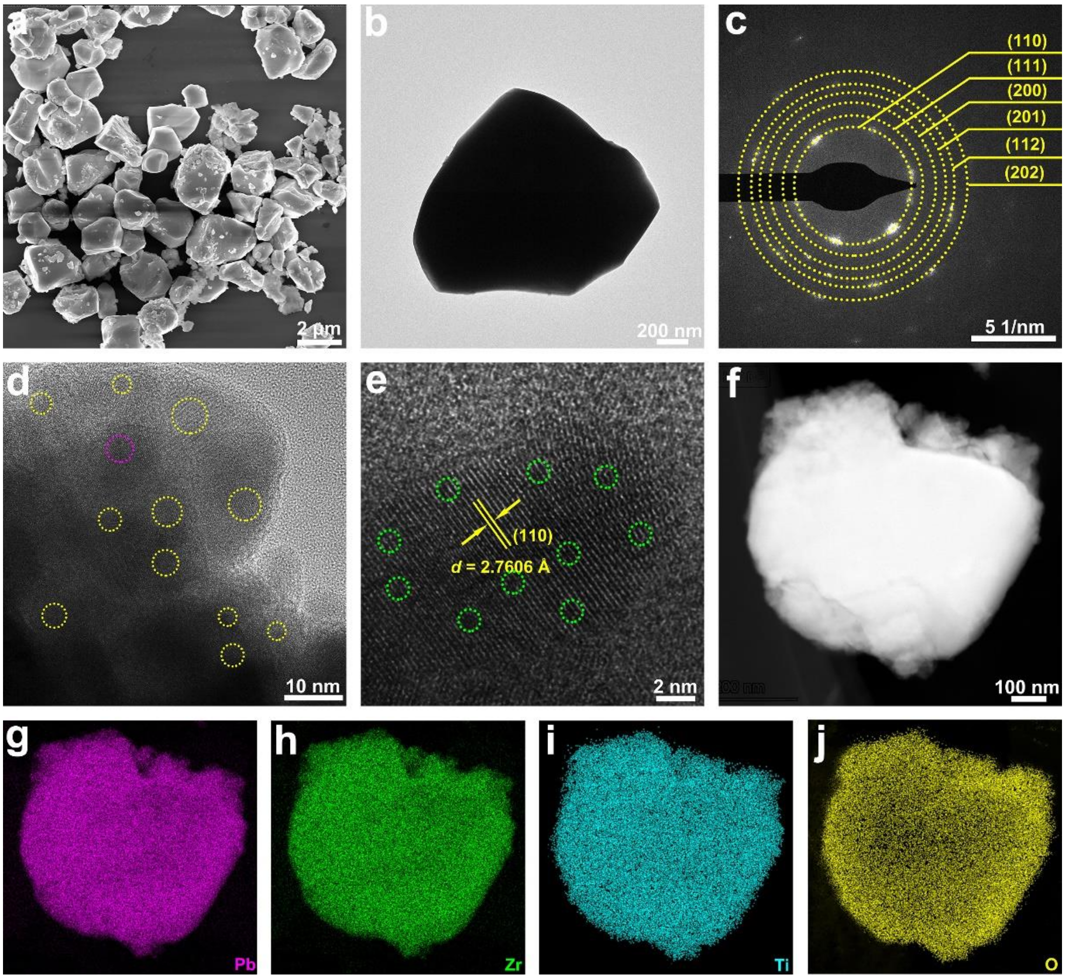

Figure S24. (a) FESEM image of $Pb(ZrTi)_{1/2}O_3$ after chronoamperometry test; (b) TEM image of $Pb(ZrTi)_{1/2}O_3$ and (c) corresponding SAED pattern, (d) high-magnified TEM and (e) high-resolution TEM images; (f) HAADF image of representative $Pb(ZrTi)_{1/2}O_3$ particle and corresponding elemental mapping of (g) Pb, (h) Zr, (i) Ti and (j) O.

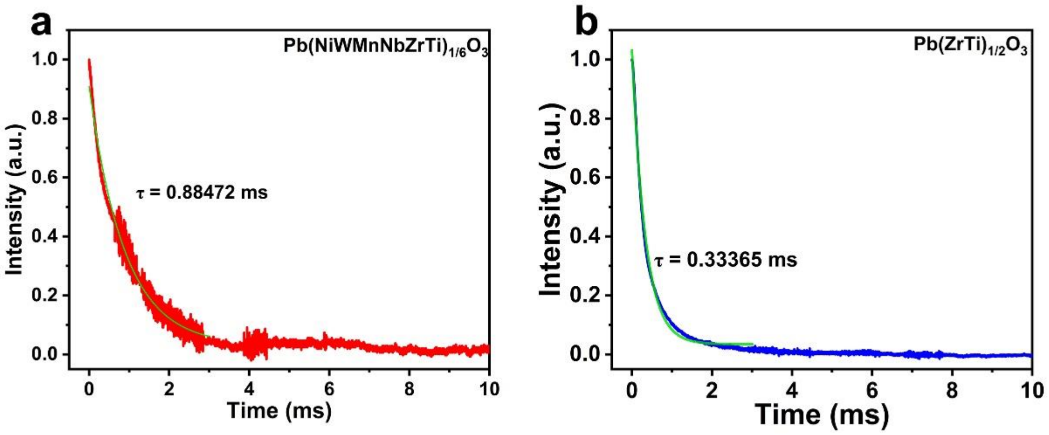

Figure S25. The fitting curves of (a) $Pb(NiWMnNbZrTi)_{1/6}O_3$ and (b) $Pb(ZrTi)_{1/2}O_3$

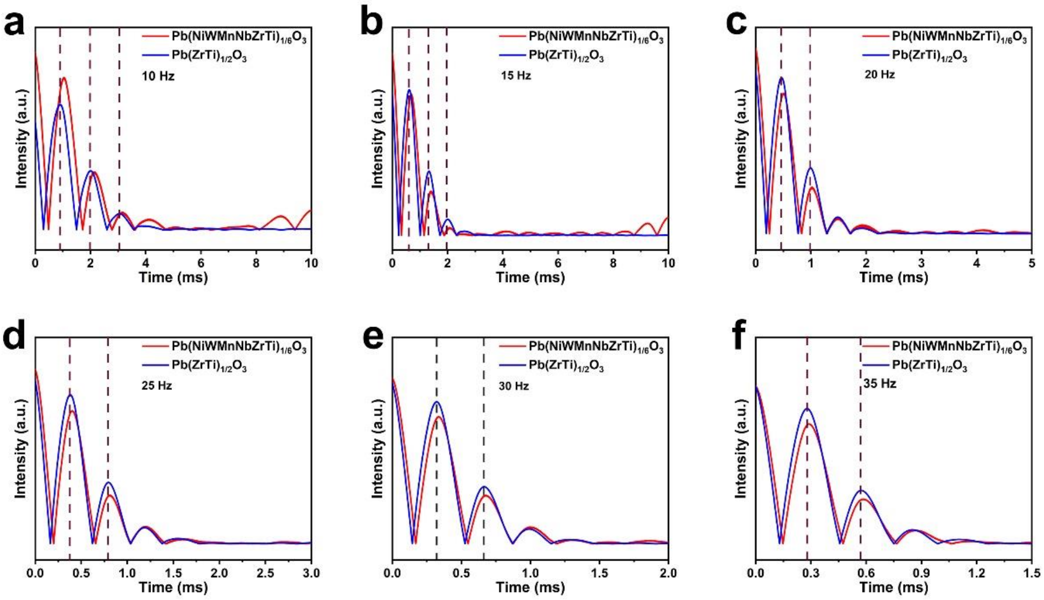

Figure S26. Comparsion of different frequencies between $Pb(NiWMnNbZrTi)_{1/6}O_3$ and $Pb(ZrTi)_{1/2}O_3$. (a) 10 Hz; (b) 15 Hz; (c) 20 Hz; (d) 25 Hz; (e) 30 Hz; (f) 35 Hz.

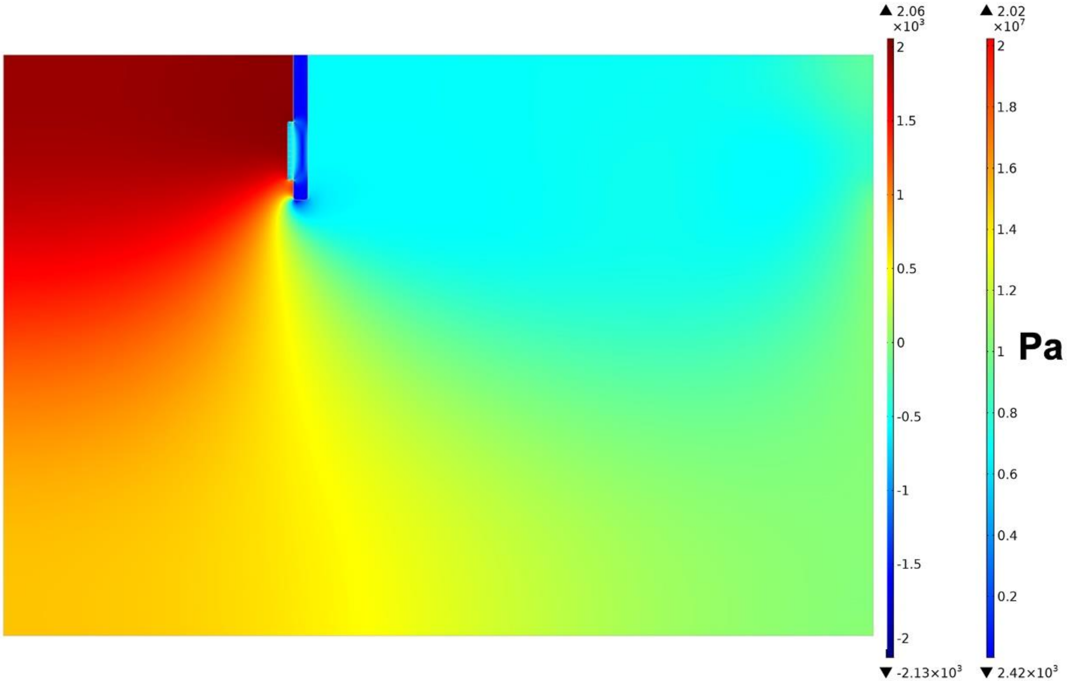

Figure S27. Stress distribution diagram of $Pb(ZrTi)_{1/2}O_3$ system under the fluid field (the left and right ruler represents the stress distribution of fluid and particle, respectively)

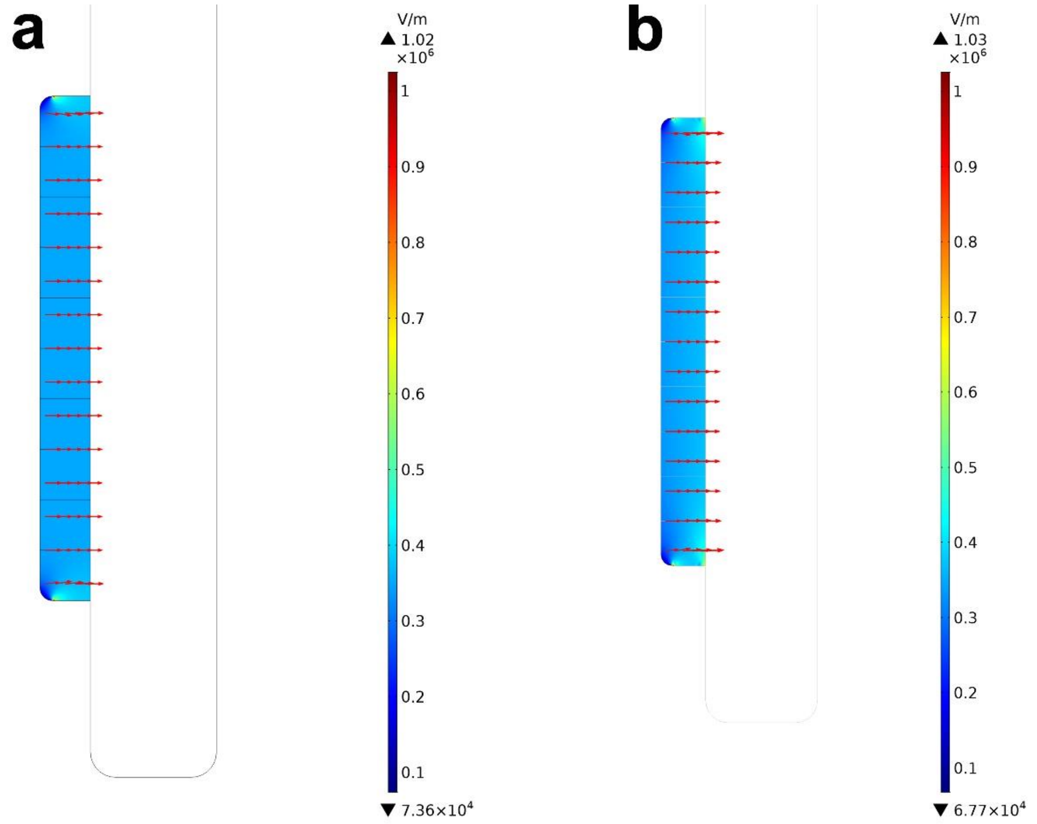

Figure S28. The surface electric field of the (a) $Pb(NiWMnNbZrTi)_{1/6}O_3$ and (b) $Pb(ZrTi)_{1/2}O_3$ particle at steady state.

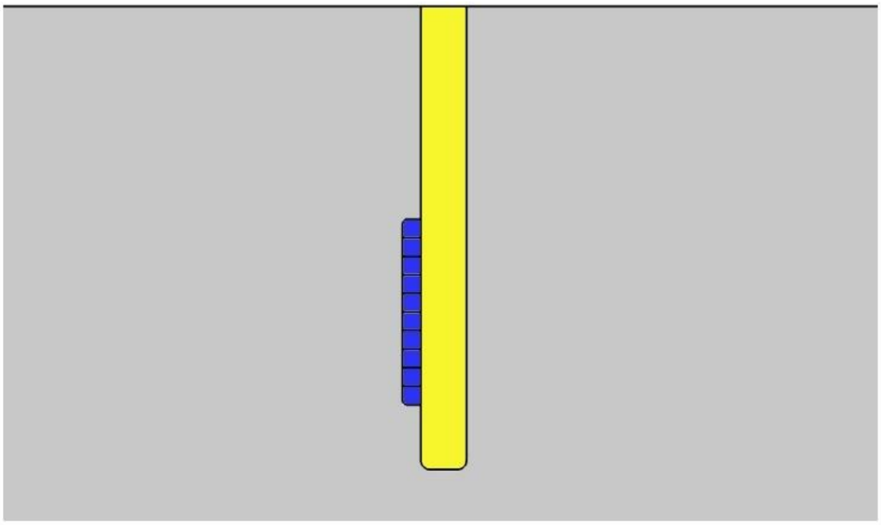

Figure S29. Simplified two-dimensional model of powder sample (blue area) deposited onto electrode (yellow area).

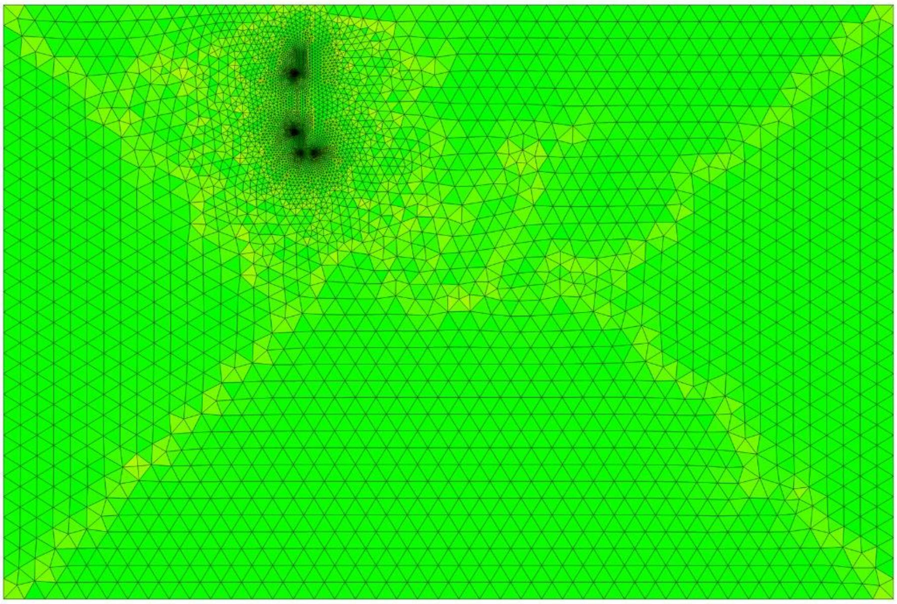

Figure S30. Meshing results by COMSOL Multiphysics® 5.5 software.

TABLES

Table S1. ICP results of $Pb(NiWMnNbZrTi)_{1/6}O_3$ and $Pb(ZrTi)_{1/2}O_3$ compounds.

Compound	Atomic ratio of metal elements (mol%)						
	Pb	Zr	Ti	Ni	W	Mn	Nb
$Pb(ZrTi)_{1/2}O_3$	1	0.54	0.46	\	\	\	\
$Pb(NiWMnNbZrTi)_{1/6}O_3$	1	0.174	0.174	0.164	0.165	0.160	0.163

Table S2. Structural parameters and phase abundance for $Pb(NiWMnNbZrTi)_{1/6}O_3$ and $Pb(ZrTi)_{1/2}O_3$ compounds refined from the experimental XRD profiles.

Sample	Space group	Lattice parameters (Å)			Abundance (wt.%)
		a	*b*	*c*	
$Pb(ZrTi)_{1/2}O_3$	*P*4*mm*	4.0358(1)	4.0358(1)	4.1434(2)	100
$Pb(NiWMnNbZrTi)_{1/6}O_3$	*Pm*-3*m*	4.0245(4)	4.0245(4)	4.0245(4)	100

Table S3. Atomic coordinates, isotropic thermal parameters (*B* values), and occupation numbers (*g* values, based on the ICP result) for the $Pb(NiWMnNbZrTi)_{1/6}O_3$ structure in the $Pb(NiWMnNbZrTi)_{1/6}O_3$ compound determined from XRD data.

atom	site	*G*	*x*	*y*	*z*	*B* (nm^2)
Pb	1*a*	1	0	0	0	0.036(1)
Ni	1*b*	0.16	0.5	0.5	0.5	0.009(3)
W	1*b*	0.16	0.5	0.5	0.5	0.001(9)
Mn	1*b*	0.16	0.5	0.5	0.5	0.002(9)
Nb	1*b*	0.16	0.5	0.5	0.5	0.163(3)
Zr	1*b*	0.18	0.5	0.5	0.5	0.002(9)
Ti	1*b*	0.18	0.5	0.5	0.5	0.006(4)
O	3*c*	1	0.5	0.5	0	0.009(2)

Table S4. Atomic coordinates, isotropic thermal parameters (*B* values), and occupation numbers (g values, based on the ICP result) for the $Pb(ZrTi)_{1/2}O_3$ structure in the $Pb(ZrTi)_{1/2}O_3$ compound determined from XRD data.

atom	site	*G*	*x*	*y*	*z*	*B* (nm^2)
Pb	1*a*	1	0	0	0	0.018(6)
Zr	1*b*	0.54	0.5	0.5	0.533(8)	0.046(1)
Ti	1*b*	0.46	0.5	0.5	0.533(8)	0.045(3)
O	2*c*	1	0.5	0	0.632(3)	0.099(3)
O	1*b*	1	0.5	0.5	0.109(6)	0.048(8)

Table S5. The comparison of ORR activity of $Pb(NiWMnNbZrTi)_{1/6}O_3$ with those of recently reported ORR catalysts in alkaline solution.

Material	Electrolyte	Selectivity	Stability	Ref.
Fe_3O_4	1 M KOH	~68 %	\	[S9]
Nb_2O_5	0.1M K_2SO_4 (pH = 2)	~72%	5.5 h	[S10]
ZrO_2	0.1M KOH	~83%	\	[S11]
TiO_2/TiC	0.1 M KOH	~90%	12 h	[S12]
$BaSm_2O_4$	0.1 M KOH	~93%	3.5h	[S13]
NiO/Fe_2O_3/NiFe-MOF	0.1 M KOH	~95%	\	[S14]
MnO_2/C	1M KOH	~91%	\	[S15]
CeO_2/C	1 M NaOH	~45%	\	[S16]
CuO_x	1M KOH	~80%	8 h	[S17]
$Sr_{0.7}Na_{0.3}Si_{0.95}Ni_{0.05}O_{3-\delta}$	0.1 M KOH	<80%	\	[S18]
Ni-LDH	0.1 M KOH	~72%	12 h	[S19]
$Ni_2Mo_4S_8$	0.1 M KOH	~92%	10 h	[S20]
$NiCo_2S_4$	0.1 M KOH	~85%	\	[S21]
$Cu_{7.2}Se_4$	0.1 M KOH	~92%	\	[S22]
Mn-N-C	0.1 M KOH	~86%	18 h	[S23]
Cu-TiO_2	0.1 M KOH	~90%	1 h	[S24]
Ni-SA/G-0	0.1 M KOH	~94%	3 h	[S25]
Pyr-2OMe	0.1 M KOH	~85%	\	[S26]
CNT@PANI-NO	0.1 M KOH	~90%	\	[S27]
N-CBMC-500	0.1 M KOH	~94%	2 h	[S28]
In SAs/NSBC	0.1 M KOH	~95%	12 h	[S29]
c-MOF Ni-150	0.1 M KOH	~95%	0.5 h	[S30]
V_2CT_x	0.1 M KOH	~89%	10 h	[S31]

CoS_2	0.1 M $HClO_4$	~46%	\	[S32]
$CoSe_2$/N-CNTs	0.1 M $HClO_4$	~92%	24 h	[S33]
$Ni_3(HITP)_2$	0.1 M KOH	~63%	\	[S34]
Fe_3N/NG	0.1 M Na_2SO_4	~66%	\	[S35]
Pd@$Au_{0.95}Pd_{0.05}$	0.1 M $HClO_4$	~90%	\	[S36]
Au–Pt–Ni	0.1 M KOH	~92%	10 h	[S37]
0.2 wt% Pt_1/TiC	0.1 M $HClO_4$	~68%	\	[S38]
0.35 wt% Pt/TiN	0.1M $HClO_4$	~56%	\	[S39]
CMK	0.5 M H_2SO_4	~81%	\	[S40]
$Pb(NiWMnNbZrTi)_{1/6}O_3$	0.1 M KOH	~96%	12 h	This work

Table S6. Elemental variation in the 0.1 M KOH electrolyte after cycle durability by ICP-OES.

Compound	Atomic ratio of metals element (ppb)						
	Pb	Zr	Ti	Ni	W	Mn	Nb
$Pb(ZrTi)_{1/2}O_3$	0.030	0.200	9.800	\	\	\	\
$Pb(NiWMnNbZrTi)_{1/6}O_3$	0.025	0.100	3.950	0.050	3.480	0.050	0.195

Table S7. The comparison of dyes degradation performance of $Pb(NiWMnNbZrTi)_{1/6}O_3$ with those of recently reported ORR catalysts.

Material	Dyes	Concentration	Time	Ref.
$CoSe_2$	Rhodamine B	20 ppm	20 min	[S41]
$CoSe_2$/N-CNT	Rhodamine B	40 ppm	40 min	[S33]
PdAu-nf	Rhodamine B	20 ppm	30 min	[S42]
B/N-HCNS@V_O-G	Rhodamine B	10 ppm	120 min	[S43]
$NiNb_2O_6$	Rhodamine B	20 ppm	20 min	[S44]
RGF	Rhodamine B	5 ppm	25 min	[S45]
O-CNTs	Rhodamine B	20 ppm	20 min	[S46]
$WO_{2.72}$/C	Orange II	30 ppm	120 min	[S47]
NiCo-MOF@GNS	Rhodamine B	25 ppm	120 min	[S48]
ZnO/h-BNC,	Methyl Orange	30 ppm	30 min	[S49]
PPy/lig-GF	Acid Orange 7	10 ppm	20 min	[S50]
Mn-N-C nanorods	Methyl Orange	25 ppm	20 min	[S23]
Fe_3N	Rhodamine B	10 ppm	60 min	[S35]
N-rGO	Methylene Blue	11 ppm	160 min	[S51]
$Pb(NiWMnNbZrTi)_{1/6}O_3$	Rhodamine B	30 ppm	15 min	This work
$Pb(NiWMnNbZrTi)_{1/6}O_3$	Methyl Orange	30 ppm	15 min	This work
$Pb(NiWMnNbZrTi)_{1/6}O_3$	Methylene Blue	30 ppm	15 min	This work

Table S8. Specific parameters of fluid field.

Steady-state average velocity at inlet	8.38 m s^{-1}
Channel height	$2*10^{-4}$ m
Channel width	$3*10^{-4}$ m
Radius of rotation	0.05 m
Rotating speed	1600 r min^{-1}

References

[S1] a) B. Ravel, M. Newville, *J. Synchrotron Radiat.* **2005**, *12*, 537-541; b) F. Izumi, K. Momma, *Solid State Phenom.* **2007**, *130*, 15-20.

[S2] a) X. Qian, Z. Cai, M. Su, F. Li, W. Fang, Y. Li, X. Zhou, Q. Li, X. Feng, W. Li, X. Hu, X. Wang, C. Pan, Y. Song, *Adv. Mater.* **2018**, *30*, e1800291; b) H. Amini, E. Sollier, M. Masaeli, Y. Xie, B. Ganapathysubramanian, H. A. Stone, D. Di Carlo, *Nat. Commun.* **2013**, *4*, 1826; c) H. Kim, S. Yun, K. Kim, W. Kim, J. Ryu, H. G. Nam, S. M. Han, S. Jeon, S. Hong, *Nano Energy* **2020**, *78*, 105259; d) Y. C. Lai, J. Deng, R. Liu, Y. C. Hsiao, S. L. Zhang, W. Peng, H. M. Wu, X. Wang, Z. L. Wang, *Adv. Mater.* **2018**, *30*, e1801114; e) R. Amin, L. F. Abdulrazak, N. Mohammadd, K. Ahmed, F. M. Bui, S. M. Ibrahim, *Ceram. Int.* **2022**, *48*, 5617-5625; f) M. Liu, Y. Pang, B. Zhang, P. De Luna, O. Voznyy, J. Xu, X. Zheng, C. T. Dinh, F. Fan, C. Cao, F. P. de Arquer, T. S. Safaei, A. Mepham, A. Klinkova, E. Kumacheva, T. Filleter, D. Sinton, S. O. Kelley, E. H. Sargent, *Nature* **2016**, *537*, 382-386.

[S3] a) Q. Li, Y. Ouyang, S. Lu, X. Bai, Y. Zhang, L. Shi, C. Ling, J. Wang, *Chem. Commun.* **2020**, *56*, 9937-9949; b) P. N. Ciesielski, M. B. Pecha, N. E. Thornburg, M. F. Crowley, X. Gao, O. Oyedeji, H. Sitaraman, N. Brunhart-Lupo, *Energy Fuels* **2021**, *35*, 14382-14400.

[S4] P. Liu, B. Chen, C. Liang, W. Yao, Y. Cui, S. Hu, P. Zou, H. Zhang, H. J. Fan, C. Yang, *Adv. Mater.* **2021**, *33*, e2007377.

[S5] Y. Wu, P. Zhai, S. Cao, Z. Li, B. Zhang, Y. Zhang, X. Nie, L. Sun, J. Hou, *Adv. Energy Mater.* **2020**, *10*, 2002499.

[S6] A. Nairan, C. Liang, S.-W. Chiang, Y. Wu, P. Zou, U. Khan, W. Liu, F. Kang, S. Guo, J. Wu, C. Yang, *Energy Environ. Sci.* **2021**, *14*, 1594-1601.

[S7] E. J. F. Dickinson, H. Ekström, E. Fontes, *Electrochem. Commun.* **2014**, *40*, 71-74.

[S8] a) R. B. Lakshmi, N. P. Harikrishnan, A. V. Juliet, *Appl. Surf. Sci.* **2017**, *418*, 99-102; b) M. Scaramuzza, A. Ferrario, E. Pasqualotto, A. De Toni, *Procedia Chem.* **2012**, *6*, 69-78.

[S9] W. R. P. Barros, Q. Wei, G. Zhang, S. Sun, M. R. V. Lanza, A. C. Tavares, *Electrochim. Acta* **2015**, *162*, 263-270.

[S10] J. F. Carneiro, M. J. Paulo, M. Siaj, A. C. Tavares, M. R. V. Lanza, *J. Catal.* **2015**, *332*, 51-61.

[S11] J. F. Carneiro, M. J. Paulo, M. Siaj, A. C. Tavares, M. R. V. Lanza, *ChemElectroChem* **2017**, *4*, 508-513.

[S12] Z. Xu, J. Liang, Y. Wang, K. Dong, X. Shi, Q. Liu, Y. Luo, T. Li, Y. Jia, A. M. Asiri, Z. Feng, Y. Wang, D. Ma, X. Sun, *ACS Appl. Mater. Interfaces* **2021**, *13*, 33182-33187.

[S13] R. Ranjan Mahalik, P. Parhi, *Mater. Today: Proc.* **2021**, *43*, 3261-3267.

[S14] M. Wang, N. Zhang, Y. Feng, Z. Hu, Q. Shao, X. Huang, *Angew. Chem., Int. Ed.* **2020**, *59*, 14373-14377.

[S15] L. R. Aveiro, A. G. M. da Silva, V. S. Antonin, E. G. Candido, L. S. Parreira, R. S. Geonmonond, I. C. de Freitas, M. R. V. Lanza, P. H. C. Camargo, M. C. Santos, *Electrochim. Acta* **2018**, *268*, 101-110.

[S16] M. H. M. T. Assumpção, A. Moraes, R. F. B. De Souza, M. L. Calegaro, M. R. V. Lanza, E. R. Leite, M. A. L. Cordeiro, P. Hammer, M. C. Santos, *Electrochim. Acta* **2013**, *111*, 339-343.

[S17] H. Xiao, B. Li, M. Zhao, Y. Li, T. Hu, J. Jia, H. Wu, *Chem. Commun.* **2021**, *57*, 4118-4121.

[S18] S. Thundiyil, S. Kurungot, R. N. Devi, *ACS Appl. Mater. Interfaces* **2021**, *13*, 382-390.

[S19] J. Huang, J. Chen, C. Fu, P. Cai, Y. Li, L. Cao, W. Liu, P. Yu, S. Wei, Z. Wen, J. Li, *ChemSusChem* **2020**, *13*, 1496-1503.

[S20] F. Xia, B. Li, Y. Liu, Y. Liu, S. Gao, K. Lu, J. Kaelin, R. Wang, T. J. Marks, Y. Cheng, *Adv. Funct. Mater.* **2021**, *31*, 2104716.

[S21] X. Xu, X. Yin, J. Fu, D. Ke, *Chem. - Eur. J.* **2021**, *27*, 14451-14460.

[S22] Q. Yuan, J. Zhao, D. H. Mok, Z. Zheng, Y. Ye, C. Liang, L. Zhou, S. Back, K. Jiang, *Nano Lett.* **2022**, *22*, 1257-1264.

[S23] J. Biemolt, K. van der Veen, N. J. Geels, G. Rothenberg, N. Yan, *Carbon* **2019**, *155*, 643-649.

[S24] Z. Deng, L. Li, Y. Ren, C. Ma, J. Liang, K. Dong, Q. Liu, Y. Luo, T. Li, B. Tang, Y. Liu, S. Gao, A. M. Asiri, S. Yan, X. Sun, *Nano Res.* **2022**, https://doi.org/10.1007/s12274-021-3995-6.

[S25] X. Song, N. Li, H. Zhang, L. Wang, Y. Yan, H. Wang, L. Wang, Z. Bian, *ACS Appl. Mater. Interfaces* **2020**, *12*, 17519-17527.

[S26] X. Yan, D. Li, L. Zhang, X. Long, D. Yang, *Appl. Catal., B* **2022**, *304*, 120908.

[S27] J. Hu, S.-S. Li, J.-F. Li, Y.-L. Wang, X.-Y. Zhang, J.-B. Chen, S.-Q. Li, L.-N. Gu, P. Chen, *Chem. Eng. J.* **2022**, *431*, 133921.

[S28] Z. Bao, J. Zhao, S. Zhang, L. Ding, X. Peng, G. Wang, Z. Zhao, X. Zhong, Z. Yao, J. Wang, *J. Mater. Chem. A* **2022**, https://doi.org/10.1039/D1TA09915A.

[S29] E. Zhang, L. Tao, J. An, J. Zhang, L. Meng, X. Zheng, Y. Wang, N. Li, S. Du, J. Zhang, D. Wang, Y. Li, *Angew. Chem., Int. Ed.* **2022**, https://doi.org/10.1002/ange.202117347.

[S30] H. Wu, T. He, M. Dan, L. Du, N. Li, Z.-Q. Liu, *Chem. Eng. J.* **2022**, *435*, 134863.

[S31] X. Huang, M. Song, J. Zhang, J. Zhang, W. Liu, C. Zhang, W. Zhang, D. Wang, *Nano Res.* **2022**, https://doi.org/10.1007/s12274-021-4057-9.

[S32] W.-W. Zhao, P. Bothra, Z. Lu, Y. Li, L.-P. Mei, K. Liu, Z. Zhao, G. Chen, S. Back, S. Siahrostami, A. Kulkarni, J. K. Nørskov, M. Bajdich, Y. Cui, *ACS Appl. Energy Mater.* **2019**, *2*, 8605-8614.

[S33] L. Zhang, J. Liang, L. Yue, Z. Xu, K. Dong, Q. Liu, Y. Luo, T. Li, X. Cheng, G. Cui, B. Tang, A. A. Alshehri, K. A. Alzahrani, X. Guo, X. Sun, *Nano Res.* **2021**, *15*, 1-6.

[S34] E. M. Miner, T. Fukushima, D. Sheberla, L. Sun, Y. Surendranath, M. Dinca, *Nat. Commun.* **2016**, *7*, 10942.

[S35] J. Xiao, J. Chen, Z. Ou, J. Lai, T. Yu, Y. Wang, *Chin. J. Catal.* **2021**, *42*, 953-962.

[S36] Y. Zhang, Z. Lyu, Z. Chen, S. Zhu, Y. Shi, R. Chen, M. Xie, Y. Yao, M. Chi, M. Shao, Y. Xia, *Angew. Chem., Int. Ed.* **2021**, *60*, 19643-19647.

[S37] Z. Zheng, Y. H. Ng, D. W. Wang, R. Amal, *Adv. Mater.* **2016**, *28*, 9949-9955.

[S38] S. Yang, Y. J. Tak, J. Kim, A. Soon, H. Lee, *ACS Catal.* **2017**, *7*, 1301-1307.

[S39] S. Yang, J. Kim, Y. J. Tak, A. Soon, H. Lee, *Angew. Chem., Int. Ed.* **2016**, *55*, 2058-2062.

[S40] Y. Sun, I. Sinev, W. Ju, A. Bergmann, S. Dresp, S. Kühl, C. Spöri, H. Schmies, H. Wang, D. Bernsmeier, B. Paul, R. Schmack, R. Kraehnert, B. Roldan Cuenya, P. Strasser, *ACS Catal.* **2018**, *8*, 2844-2856.

[S41] H. Sheng, A. N. Janes, R. D. Ross, D. Kaiman, J. Huang, B. Song, J. R. Schmidt, S. Jin, *Energy Environ. Sci.* **2020**, *13*, 4189-4203.

[S42] X. Zhao, H. Yang, J. Xu, T. Cheng, Y. Li, *ACS Mater. Lett.* **2021**, *3*, 996-1002.

[S43] Y. Wu, Z. Gao, Y. Feng, Q. Cui, C. Du, C. Yu, L. Liang, W. Zhao, J. Feng, J. Sun, R. Yang, J. Sun, *Appl. Catal., B* **2021**, *298*, 120572.

[S44] C. Liu, H. Li, J. Chen, Z. Yu, Q. Ru, S. Li, G. Henkelman, L. Wei, Y. Chen, *Small* **2021**, *17*, e2007249.

[S45] Z. Pan, K. Wang, Y. Wang, P. Tsiakaras, S. Song, *Appl. Catal., B* **2018**, *237*, 392-400.

[S46] J. Wang, R. Chen, T. Zhang, J. Wan, X. Cheng, J. Zhao, X. Wang, *Ind. Eng. Chem. Res.* **2020**, *59*, 10364-10372.

[S47] E. C. Paz, L. R. Aveiro, V. S. Pinheiro, F. M. Souza, V. B. Lima, F. L. Silva, P. Hammer, M. R. V. Lanza, M. C. Santos, *Appl. Catal., B* **2018**, *232*, 436-445.

[S48] R. Palani, V. Anitha, C. Karuppiah, S. Rajalakshmi, Y. J. Li, T. F. Hung, C. C. Yang, *ACS Omega* **2021**, *6*, 16029-16042.

[S49] A. Yamuna, A. Mandalam, A. Karthigaiselvi, M. Balasubramanian, B. Thiruparasakthi, S. Ravichandran, S. Mayavan, *RSC Adv.* **2015**, *5*, 69394-69399.

[S50] H. Huang, C. Han, G. Wang, C. Feng, *Electrochim. Acta* **2018**, *259*, 637-646.

[S51] C.-Y. Chen, C. Tang, H.-F. Wang, C.-M. Chen, X. Zhang, X. Huang, Q. Zhang, *ChemSusChem* **2016**, *9*, 1194-1199.
